# Supplementary material for: One-Pot Synthesis of Phosphinylphosphonate Derivatives and Their Anti-Tumor Evaluations
Source: Molecules. 2021 Dec 15;26(24):7609. doi: 10.3390/molecules26247609 (PMC8703271; doi:10.3390/molecules26247609)
Supplement: Supplementary file 1 [file molecules-26-07609-s001.zip › molecules-1494044-supplementary.pdf]

One-pot Synthesis of Phosphinylphosphonate Derivatives and Their Anti-tumor Evaluations

Jade Dussart-Gautheret, Julia Deschamp, Thibaut Legigan, Maelle Monteil,
Evelyne Migianu-Griffoni,* and Marc Lecouvey*

Université Sorbonne Paris Nord, CSPBAT, CNRS, UMR 7244,
1 rue de Chablis F-93000 Bobigny, France

Supporting Information

NMR DATA OF 1-HYDROXYMETHYLENE-1,1-(*H*-PHOSPHINYLPHOSPHONATES) 6A-P AND 14O-P 2

NMR data of 1-hydroxymethylene-1,1-(*H*-phosphinylphosphonates) 6a-p and 14o-p

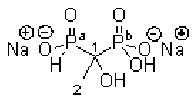

6a

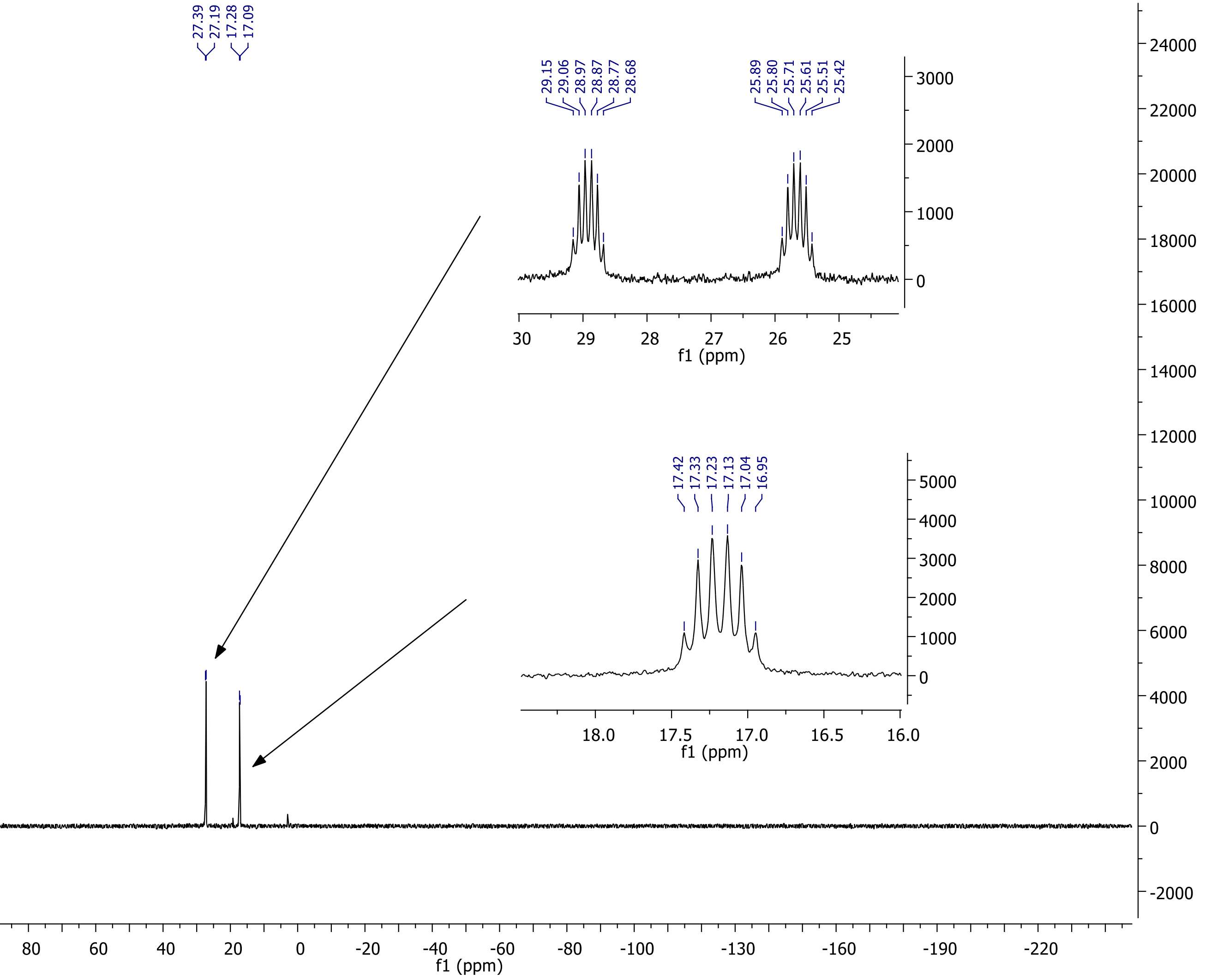

Figure S1: $^{31}P\{^{1}H\}$ and ^{31}P NMR (162 MHz, D_2O) spectra of 1-hydroxyethane-1,1-bis(*H*-phosphinylphosphonate) disodium salt 6a

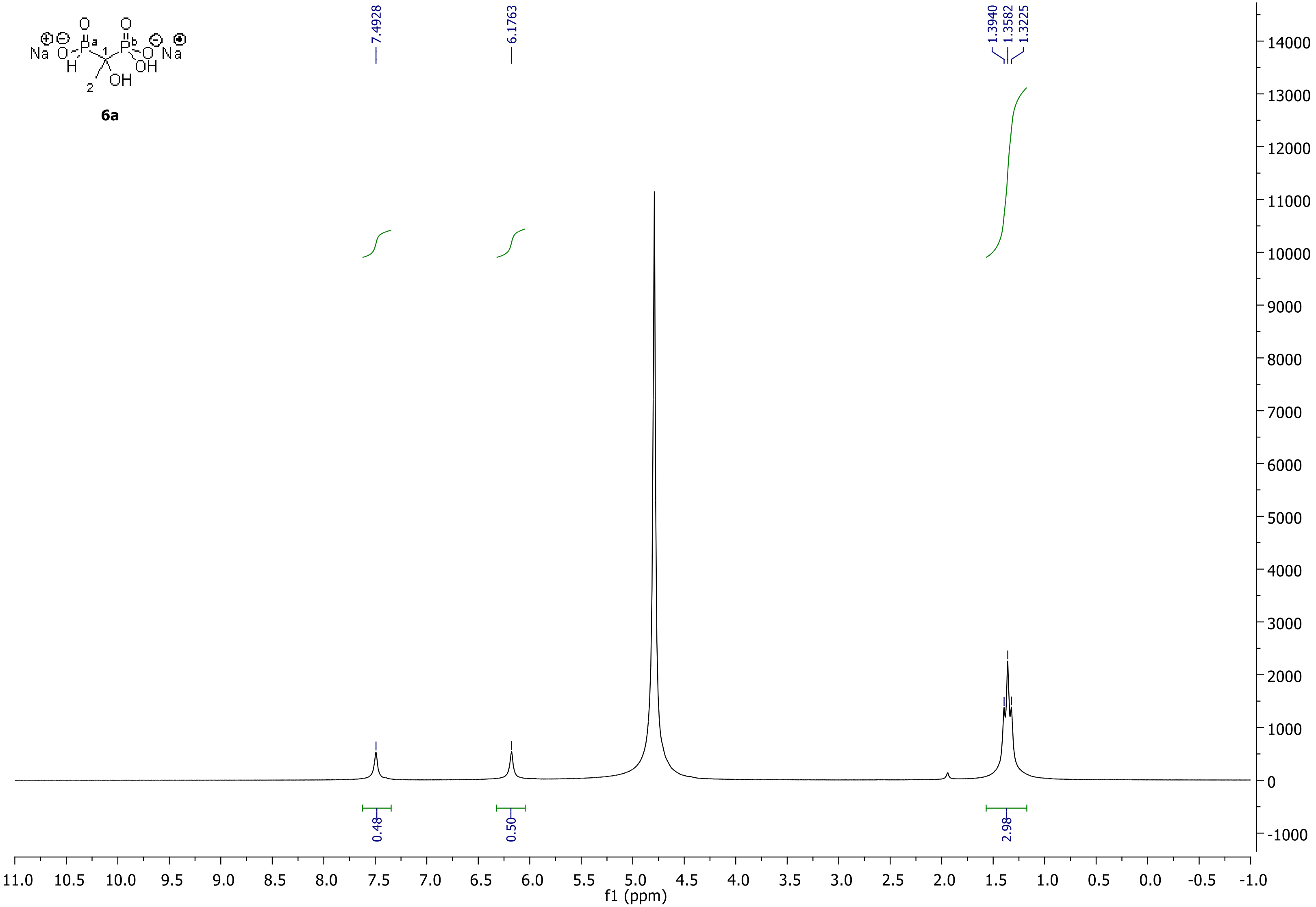

Figure S2: ^{1}H NMR (400 MHz, D_2O) spectrum of 1-hydroxyethane-1,1-bis(*H*-phosphinylphosphonate) disodium salt 6a

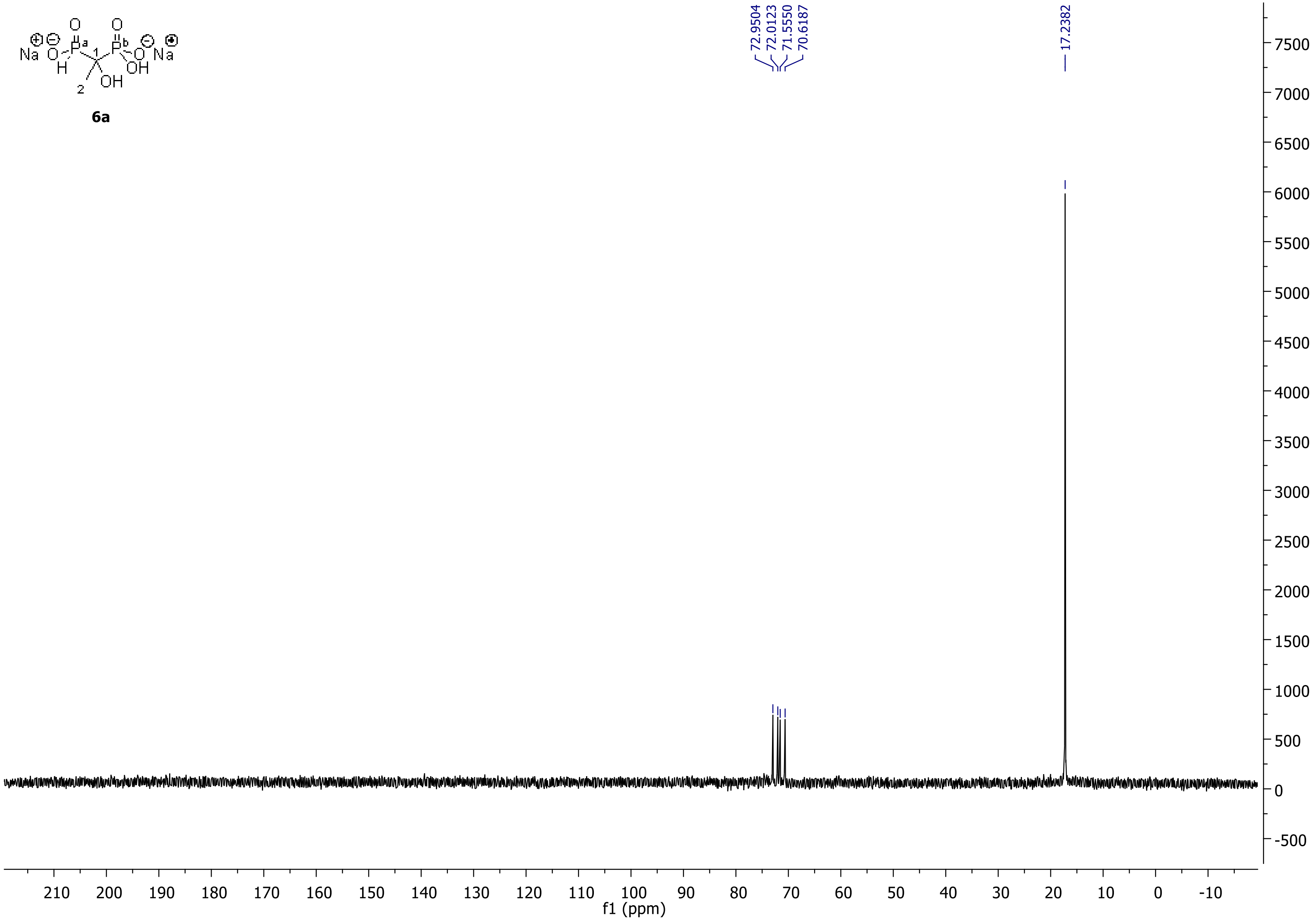

Figure S3: ^{13}C NMR (101 MHz, D_2O) spectrum of 1-hydroxyethane-1,1-bis(*H*-phosphinylphosphonate) disodium salt 6a

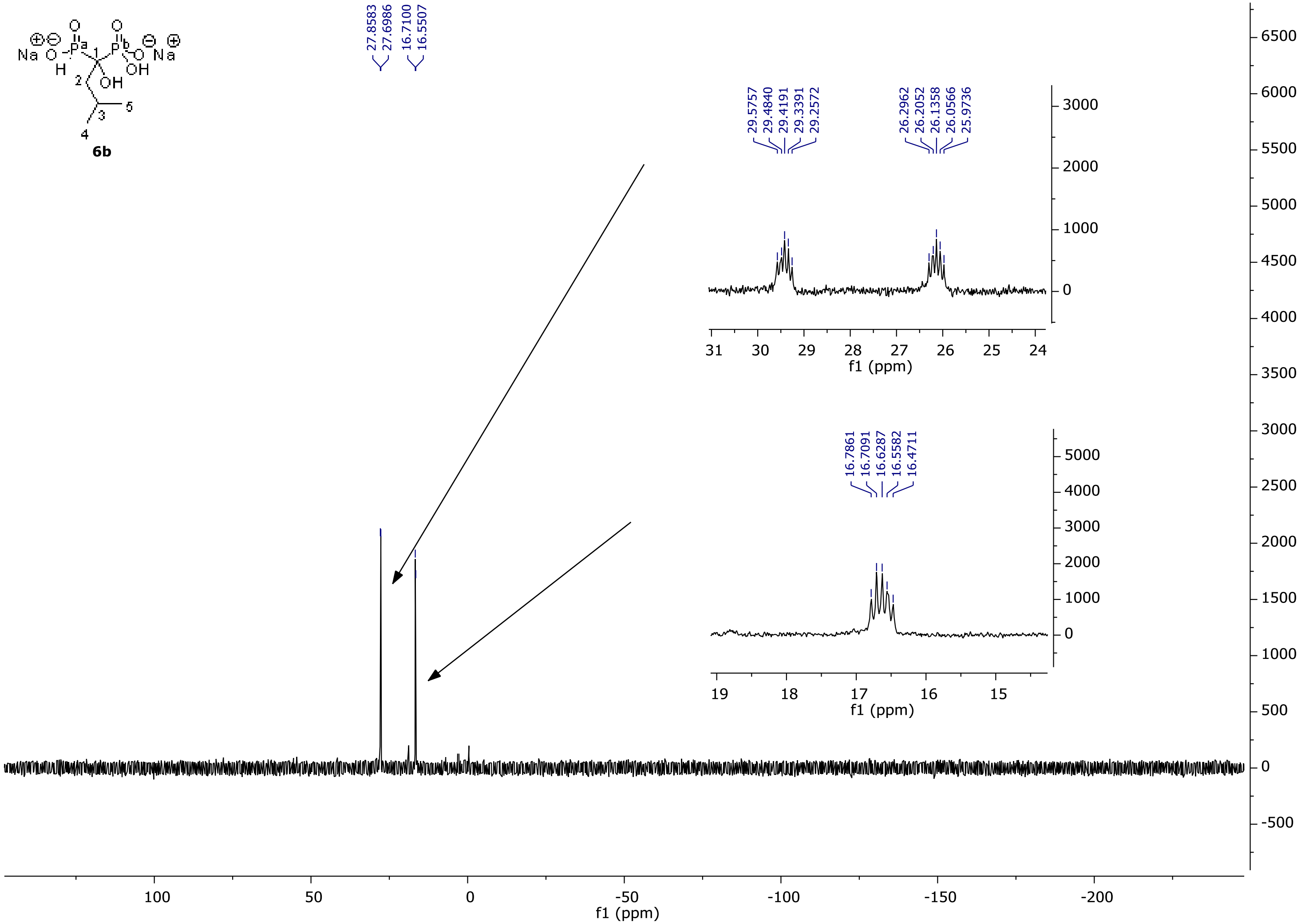

Figure S4: $^{31}P\{^{1}H\}$ and ^{31}P NMR (162 MHz, D_2O) spectra of 1-hydroxy-3-methylbutane-1,1-bis(*H*-phosphinylphosphonate) disodium salt 6b

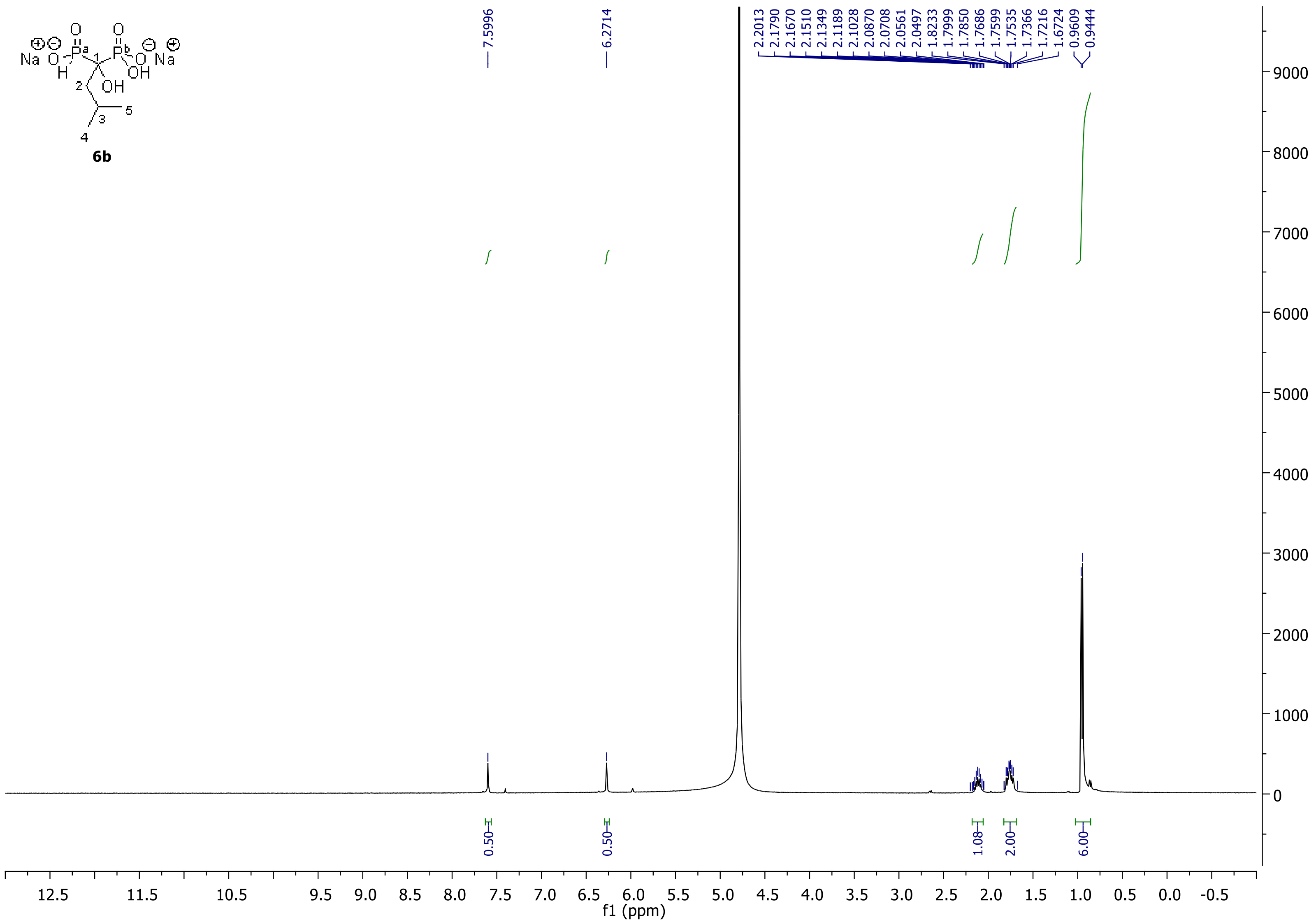

Figure S5: ^{1}H NMR (400 MHz, D_2O) spectrum of 1-hydroxy-3-methylbutane-1,1-bis(*H*-phosphinylphosphonate) disodium salt 6b

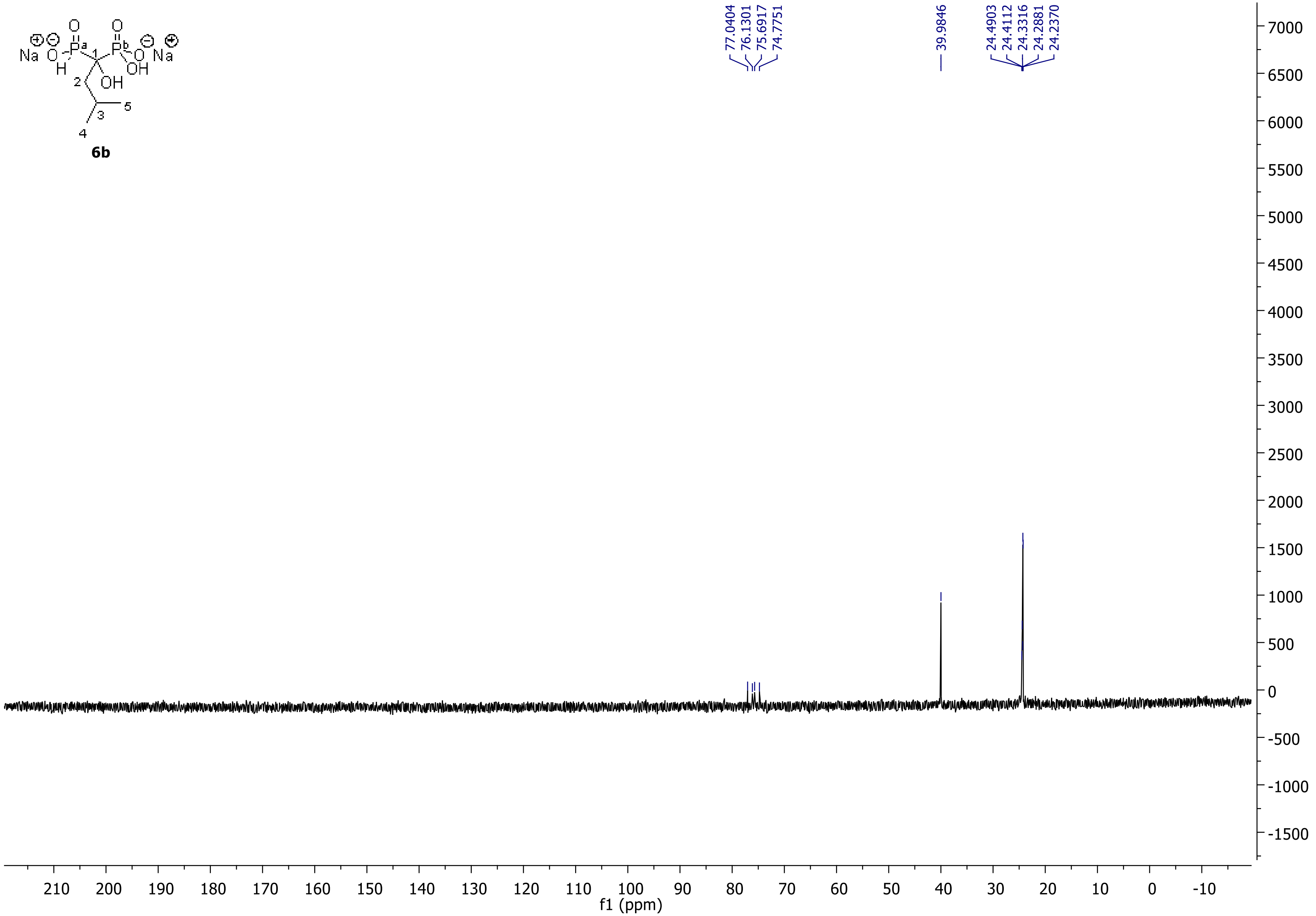

Figure S6: ^{13}C NMR (101 MHz, D_2O) spectrum of 1-hydroxy-3-methylbutane-1,1-bis(*H*-phosphinylphosphonate) disodium salt 6b

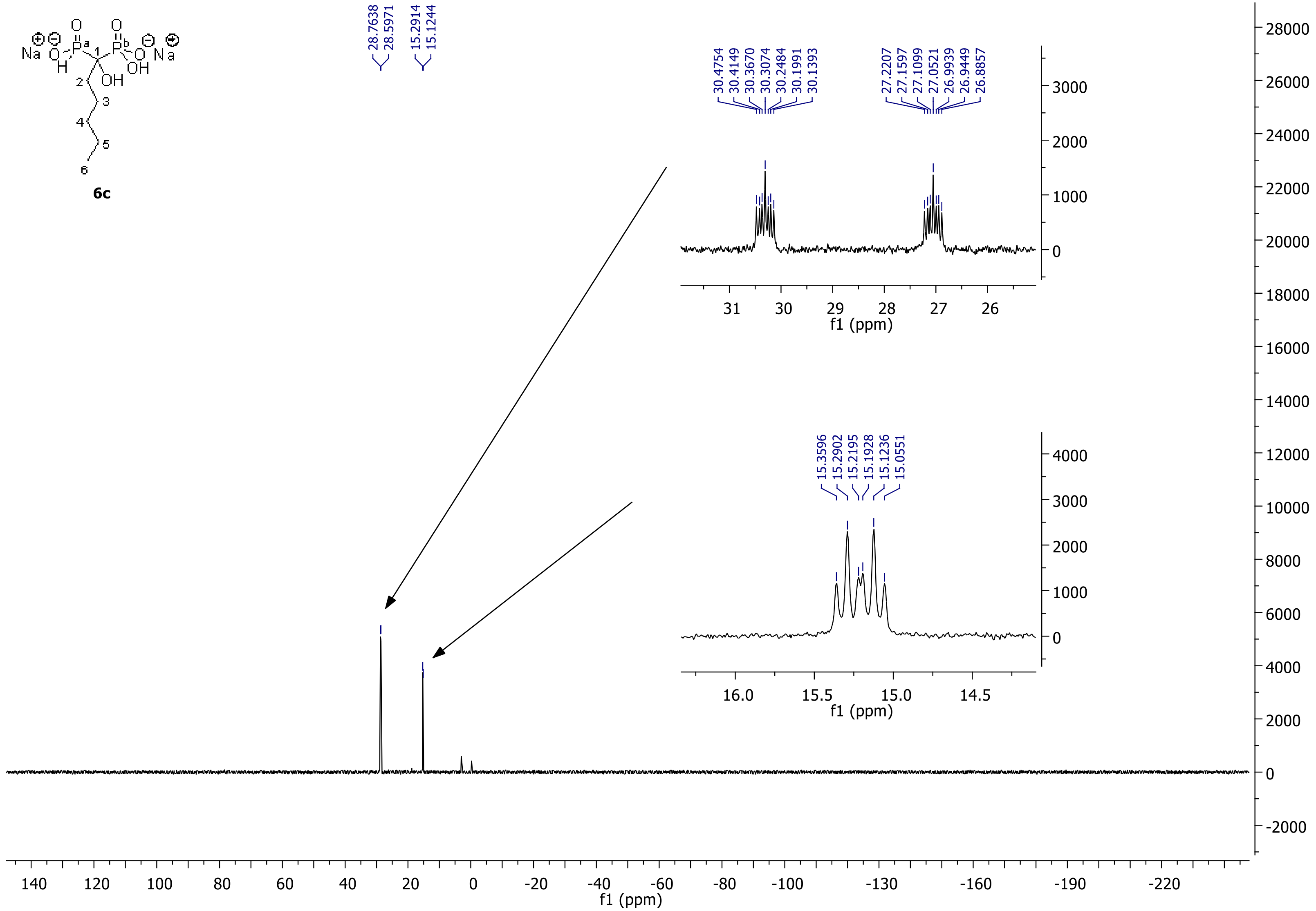

Figure S7: $^{31}P\{^{1}H\}$ and ^{31}P NMR (162 MHz, D_2O) spectra of 1-hydroxyhexane-1,1-bis(*H*-phosphinylphosphonate) disodium salt 6c

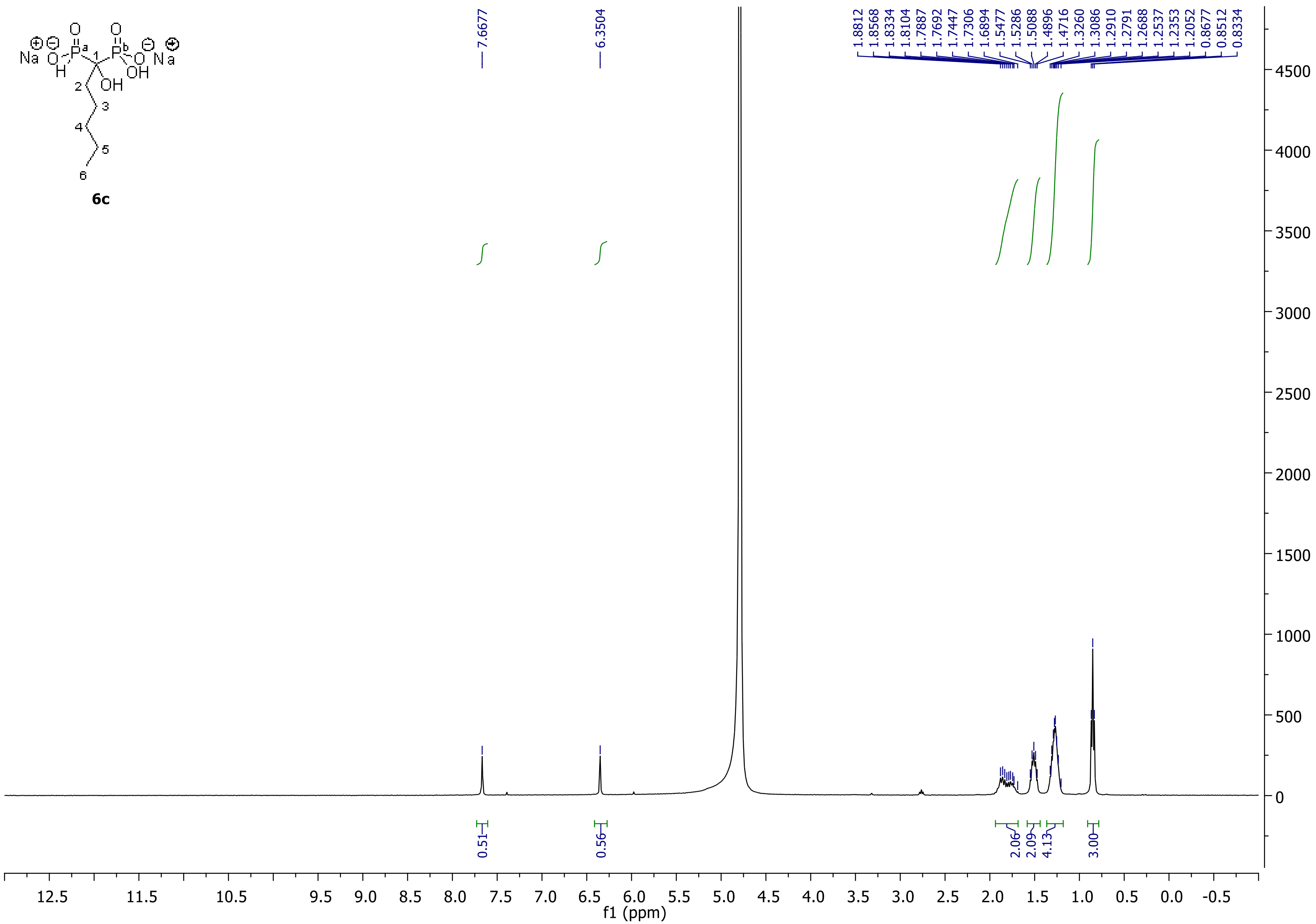

Figure S8: ^{1}H NMR (400 MHz, D_2O) spectrum of 1-hydroxyhexane-1,1-bis(*H*-phosphinylphosphonate) disodium salt 6c

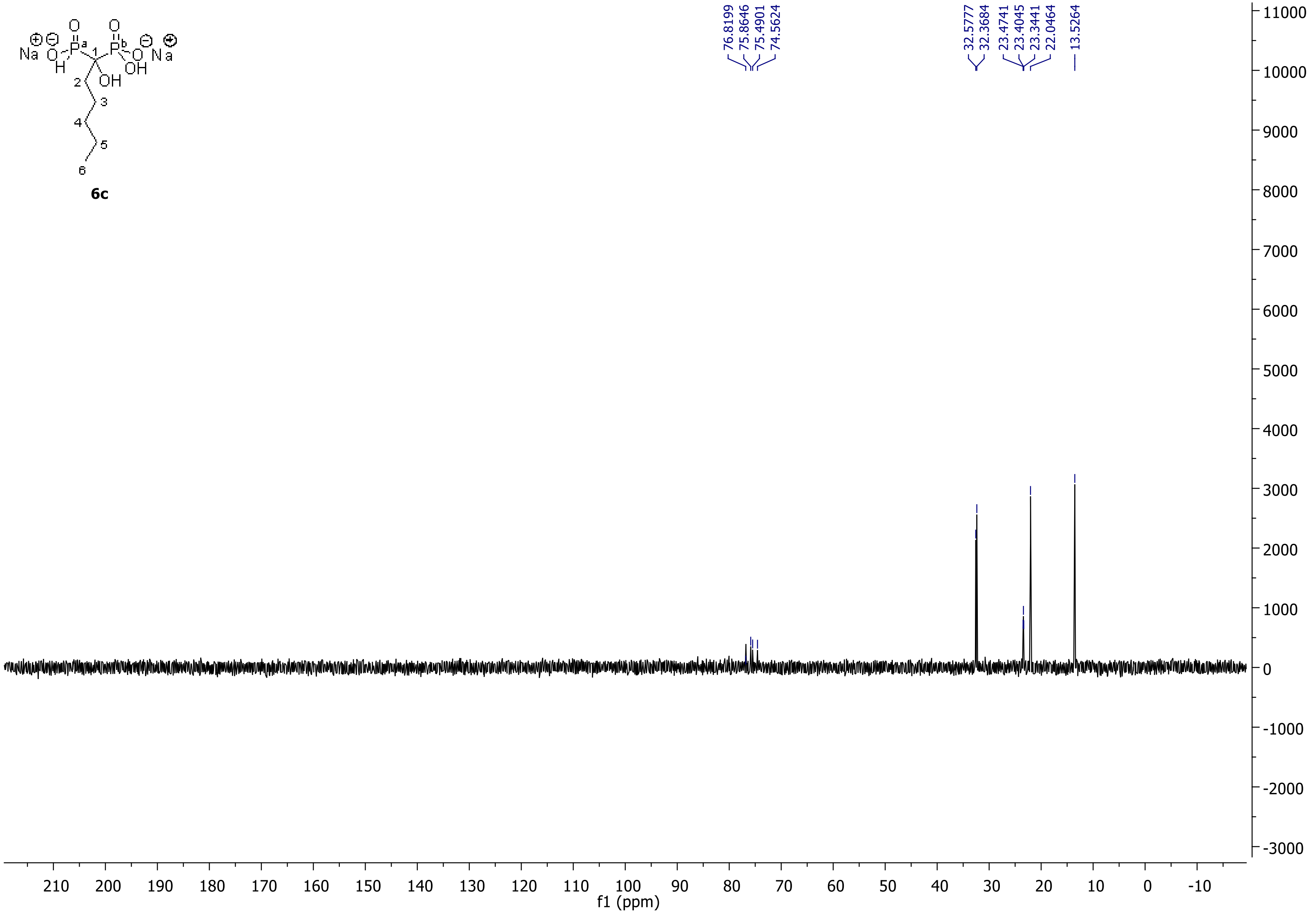

Figure S9: ^{13}C NMR (101 MHz, D_2O) spectrum of 1-hydroxyhexane-1,1-bis(*H*-phosphinylphosphonate) disodium salt 6c

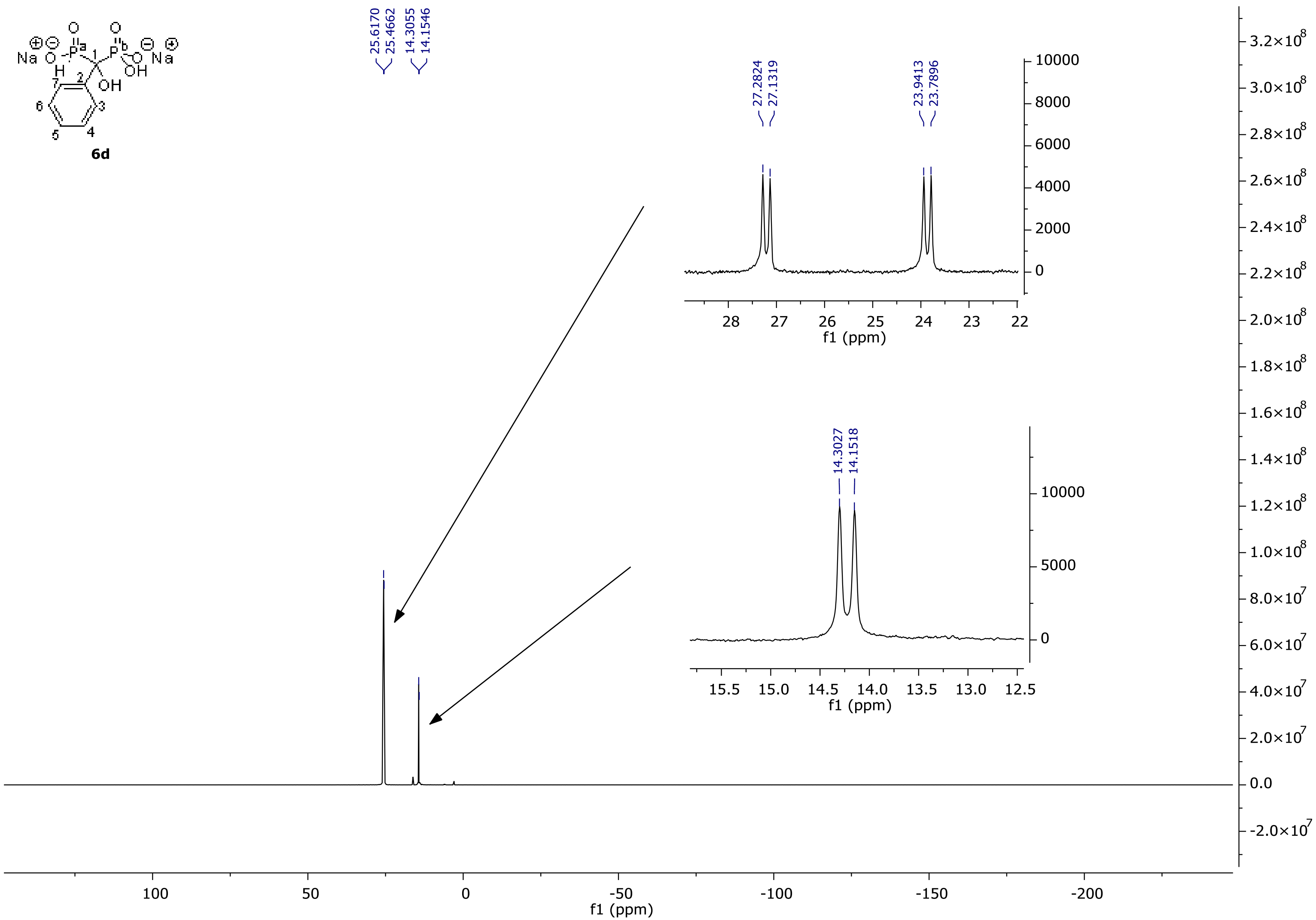

Figure S10: $^{31}P\{^{1}H\}$ and ^{31}P NMR (162 MHz, D_2O) spectra of 1-hydroxy-1-phenylmethane-1,1-bis(*H*-phosphinylphosphonate) disodium salt 6d

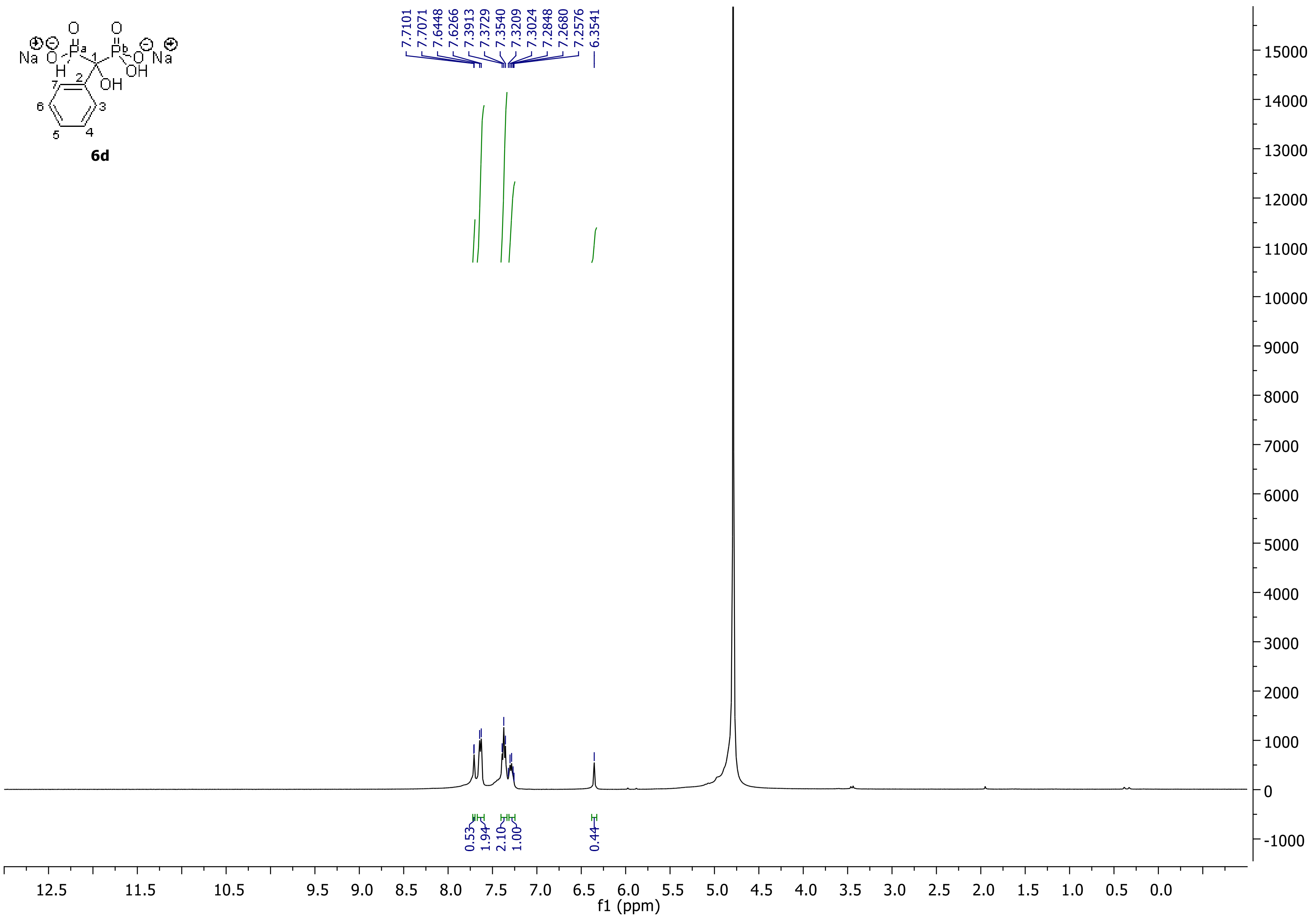

Figure S11: ^{1}H NMR (400 MHz, D_2O) spectrum of 1-hydroxy-1-phenylmethane-1,1-bis(*H*-phosphinylphosphonate) disodium salt 6d

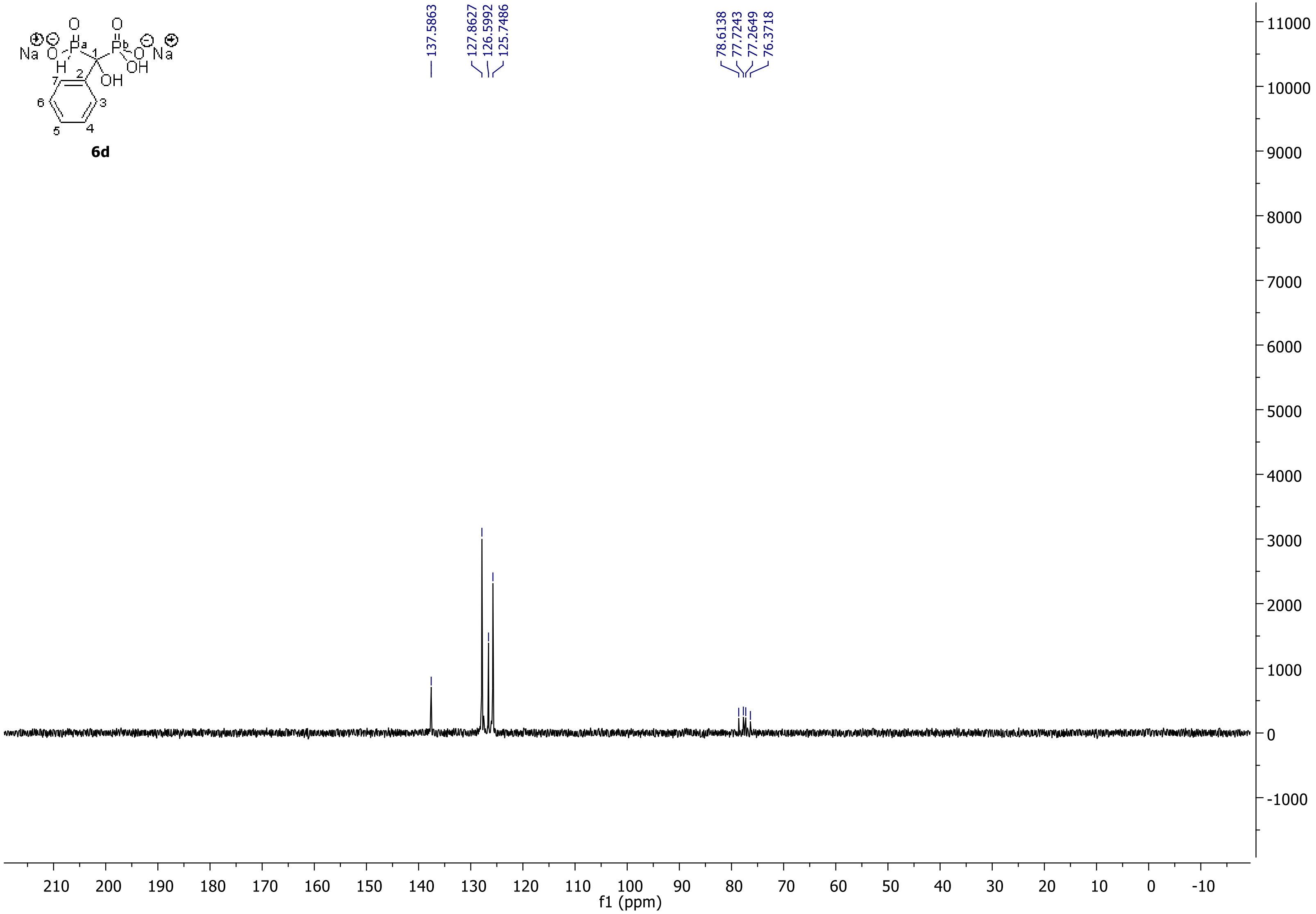

Figure S12: ^{13}C NMR (101 MHz, D_2O) spectrum of 1-hydroxy-1-phenylmethane-1,1-bis(*H*-phosphinylphosphonate) disodium salt 6d

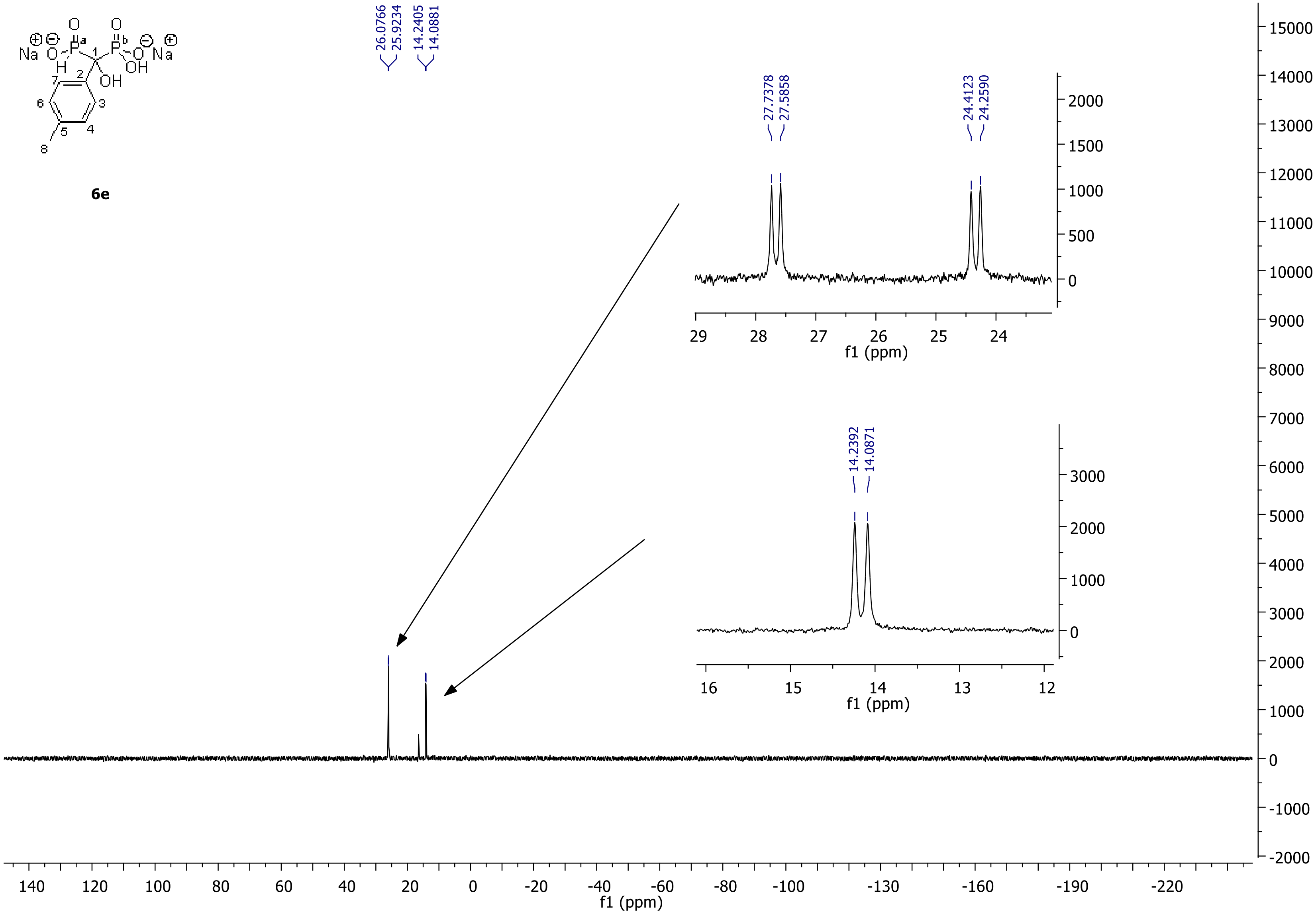

Figure S13: $^{31}P\{^{1}H\}$ and ^{31}P NMR (162 MHz, D_2O) spectra of 1-hydroxy-1-(4-tolyl)methane-1,1-bis(*H*-phosphinylphosphonate) disodium salt 6e

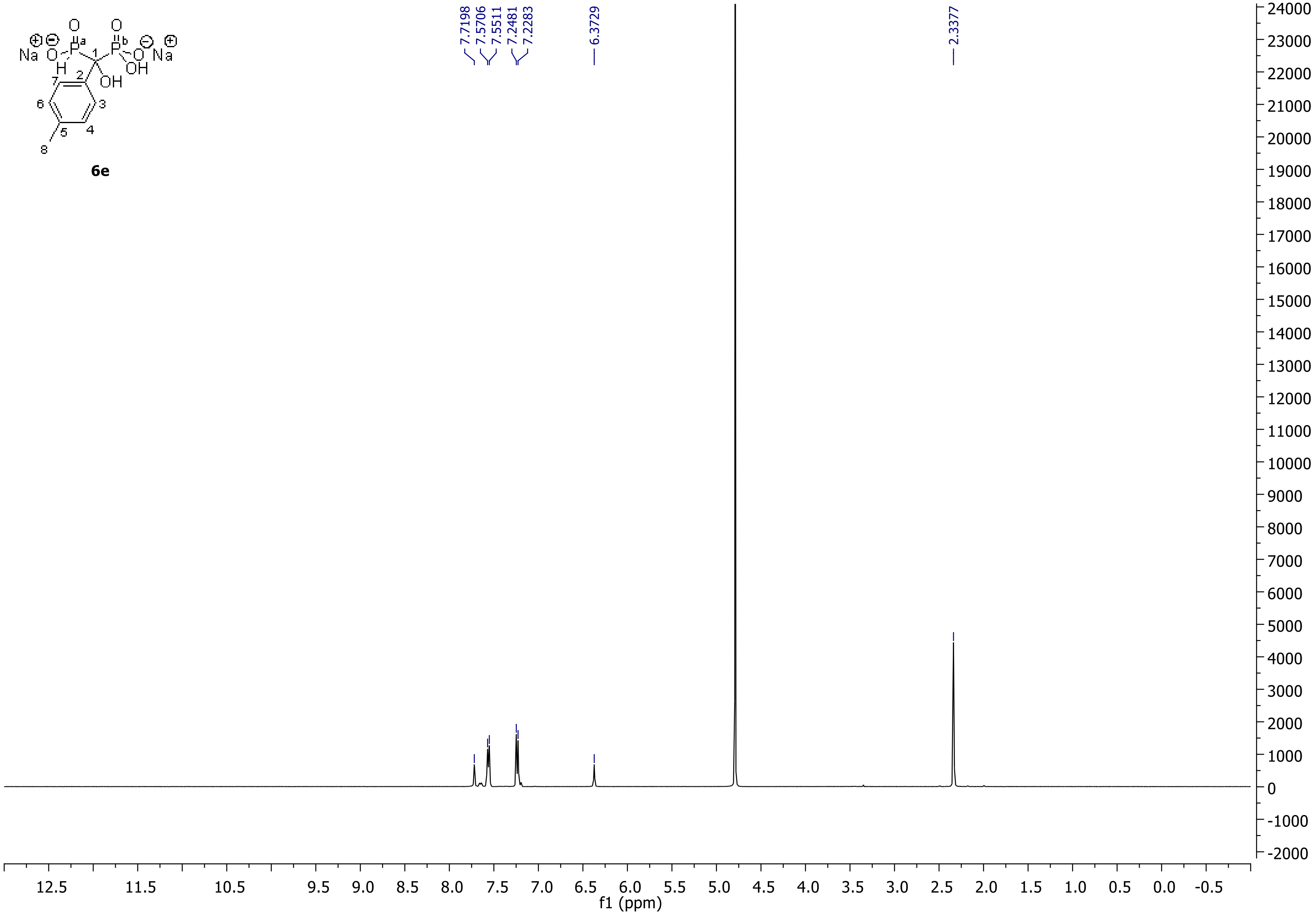

Figure S14: ^{1}H NMR (400 MHz, D_2O) spectrum of 1-hydroxy-1-(4-tolyl)methane-1,1-bis(*H*-phosphinylphosphonate) disodium salt 6e

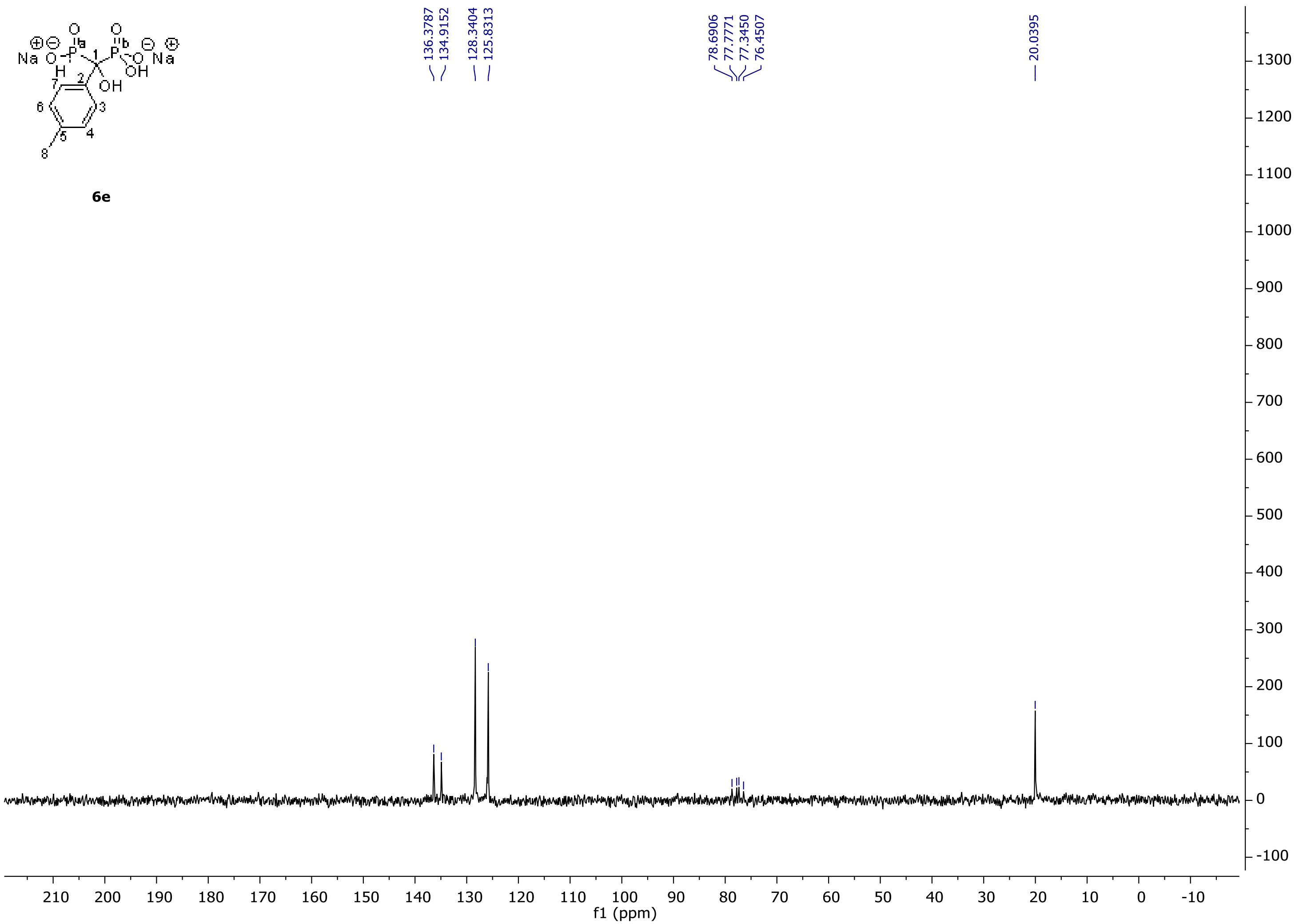

Figure S15: ^{13}C NMR (101 MHz, D_2O) spectrum of 1-hydroxy-1-(4-tolyl)methane-1,1-bis(*H*-phosphinylphosphonate) disodium salt 6e

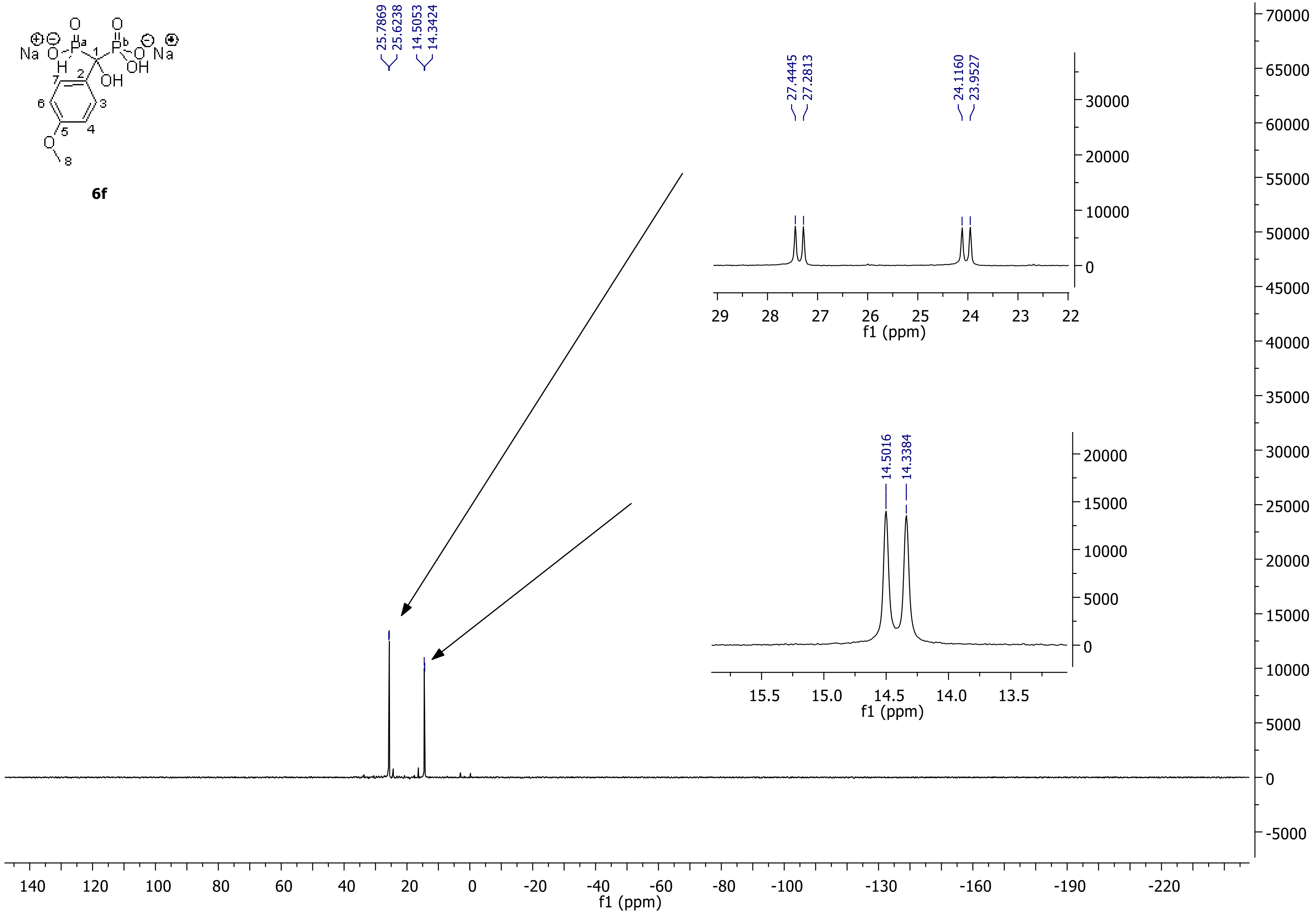

Figure S16: $^{31}P\{^{1}H\}$ and ^{31}P NMR (162 MHz, D_2O) spectra of hydroxy-1-(4-methoxyphenyl)methane-1,1-bis(*H*-phosphinylphosphonate) disodium salt 6f

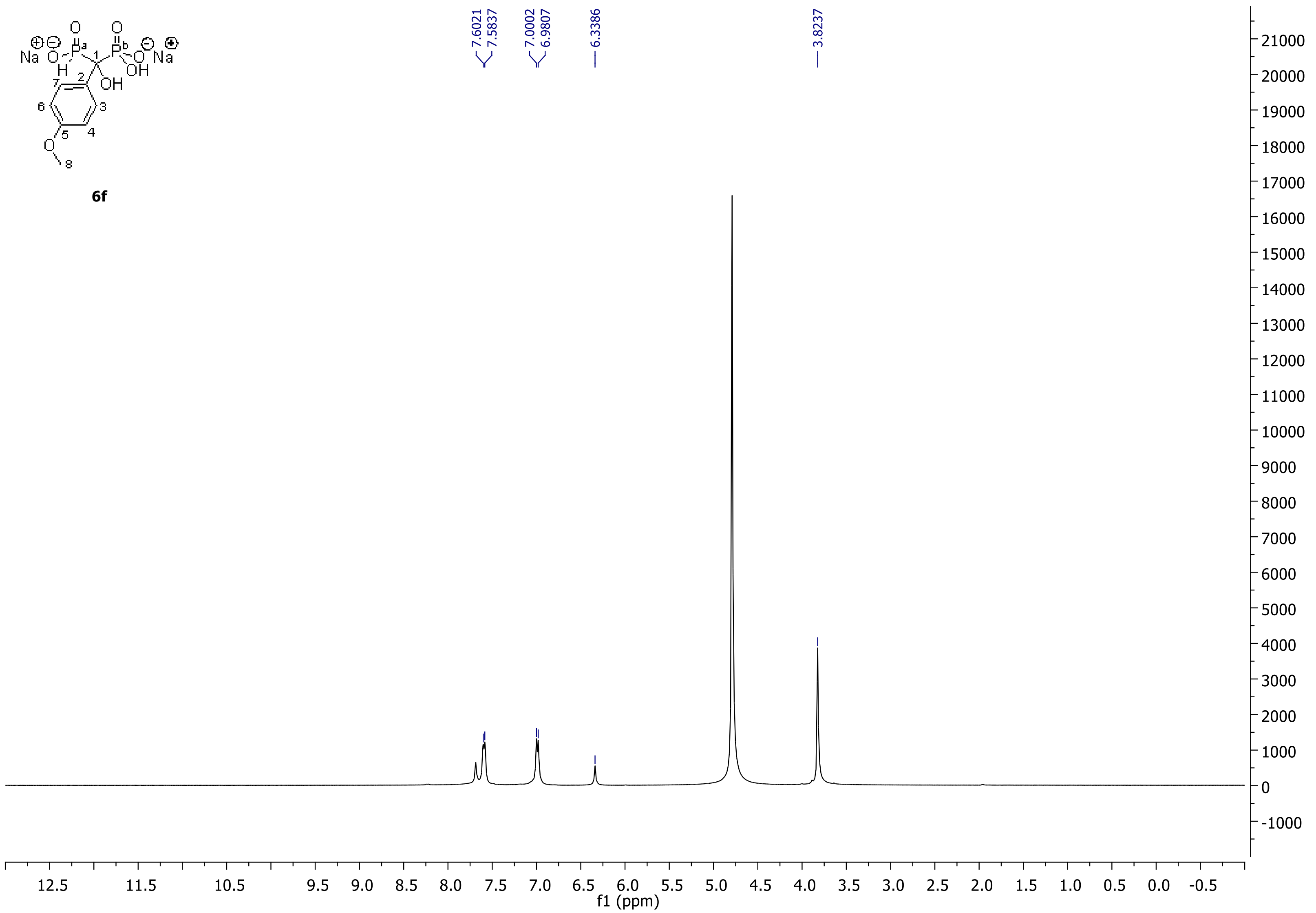

Figure S17: ^{1}H NMR (400 MHz, D_2O) spectrum of hydroxy-1-(4-methoxyphenyl)methane-1,1-bis(*H*-phosphinylphosphonate) disodium salt 6f

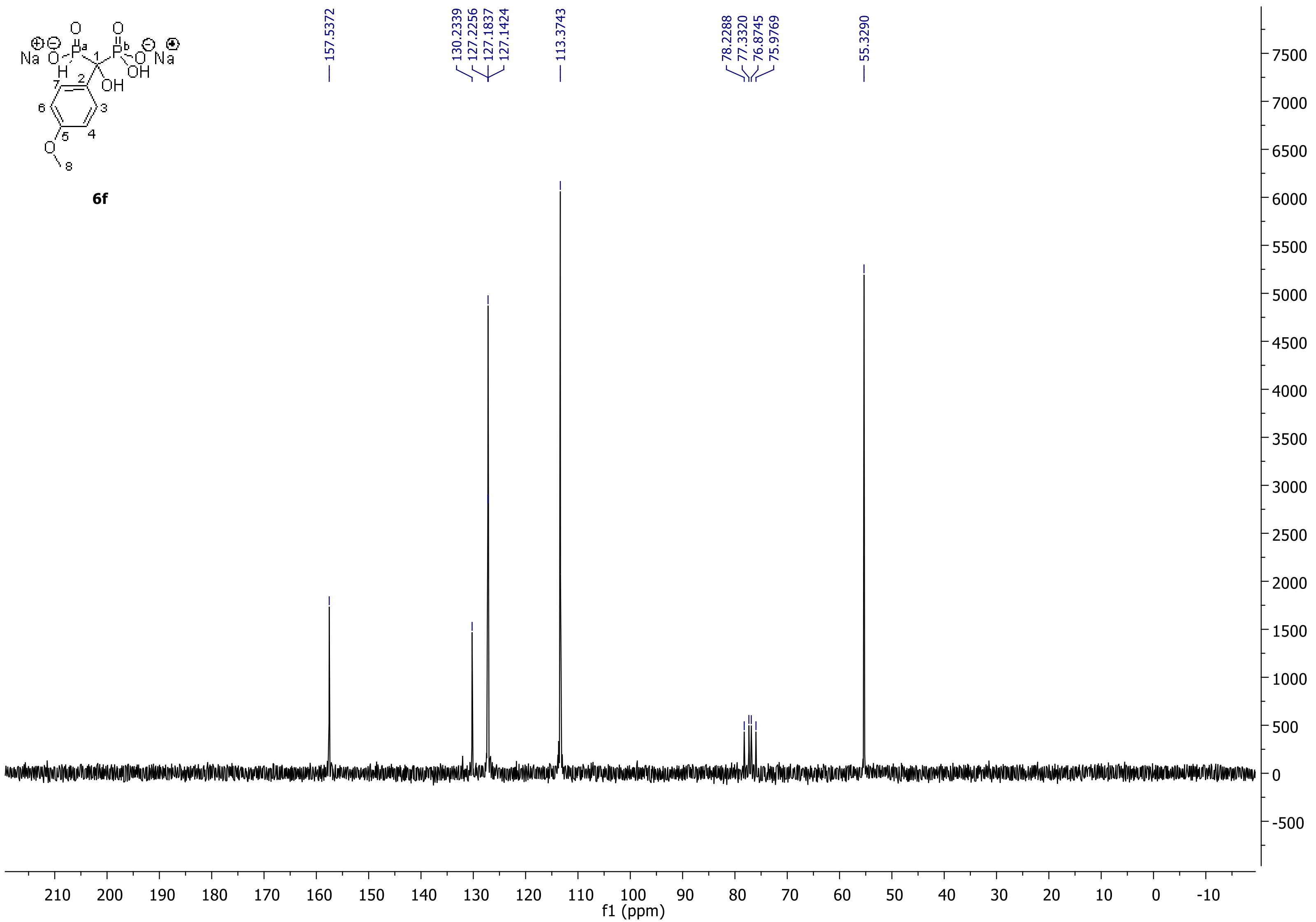

Figure S18: ^{13}C NMR (101 MHz, D_2O) spectrum of hydroxy-1-(4-methoxyphenyl)methane-1,1-bis(*H*-phosphinylphosphonate) disodium salt 6f

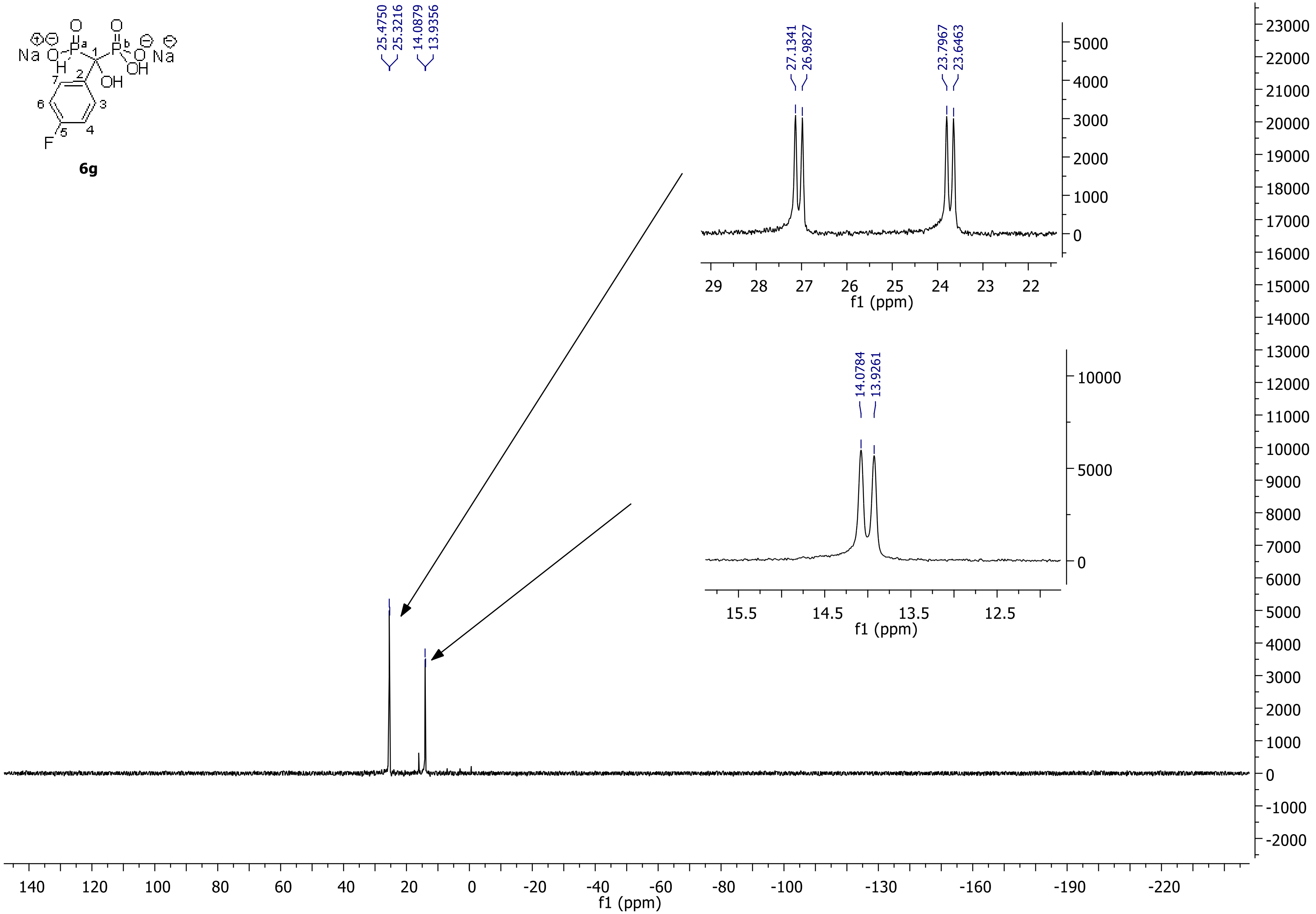

Figure S19: $^{31}P\{^1H\}$ and ^{31}P NMR (162 MHz, D_2O) spectra of 1-hydroxy-1-(4-fluorophenyl)methane-1,1-bis(*H*-phosphinylphosphonate) disodium salt 6g

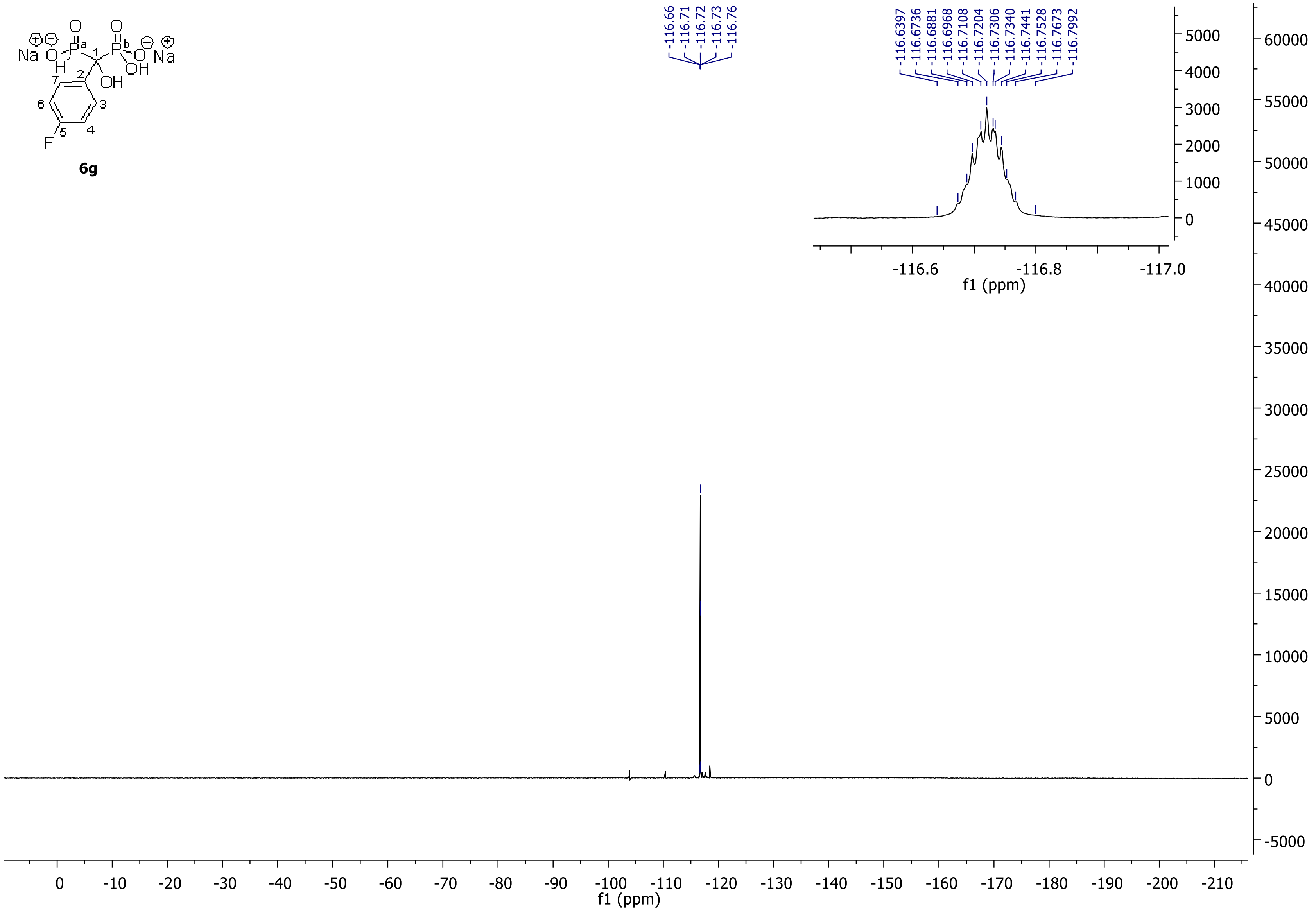

6g

Figure S20: ^{19}F {^{1}H} and ^{19}F (377 MHz, D_2O) spectra of 1-hydroxy-1-(4-fluorophenyl)methane-1,1-bis(*H*-phosphinylphosphonate) disodium salt 6g

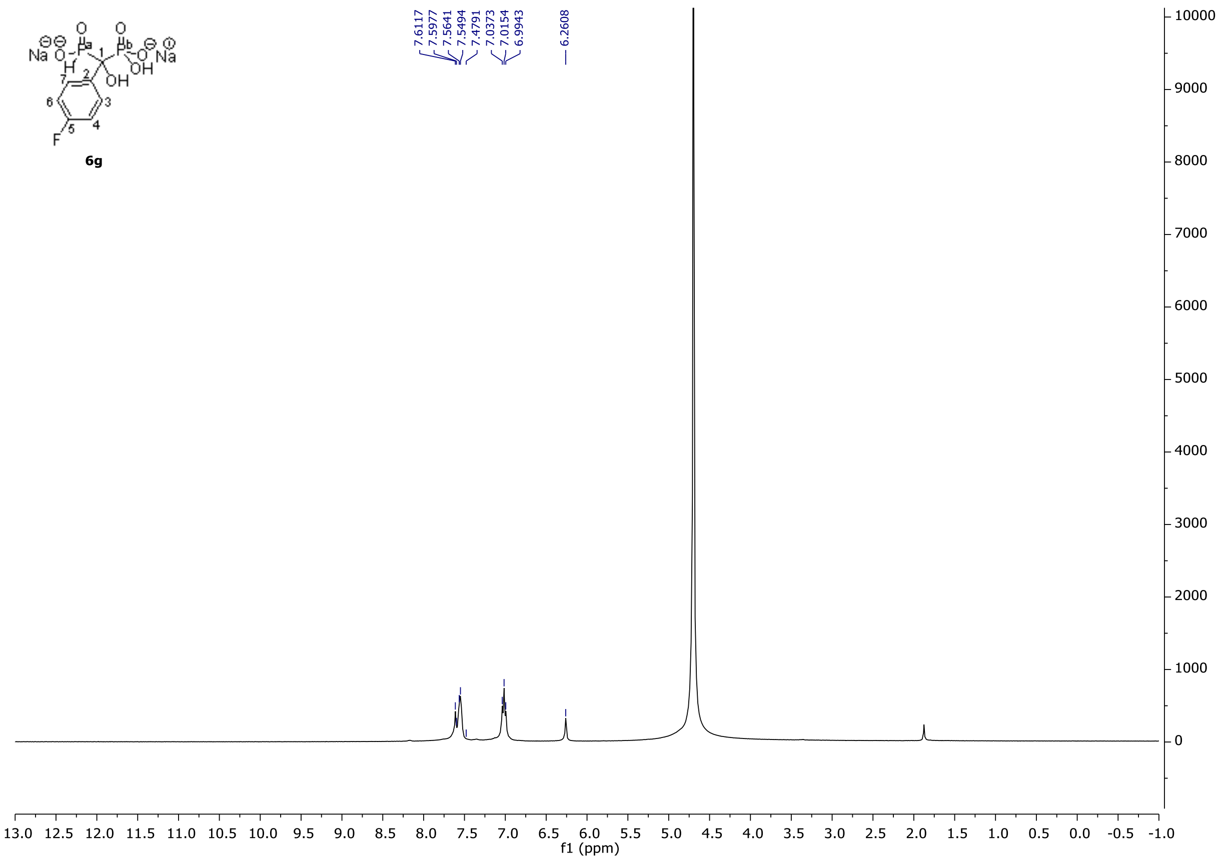

Figure S21: ^{1}H NMR (400 MHz, D_2O) spectrum of 1-hydroxy-1-(4-fluorophenyl)methane-1,1-bis(*H*-phosphinylphosphonate) disodium salt 6g

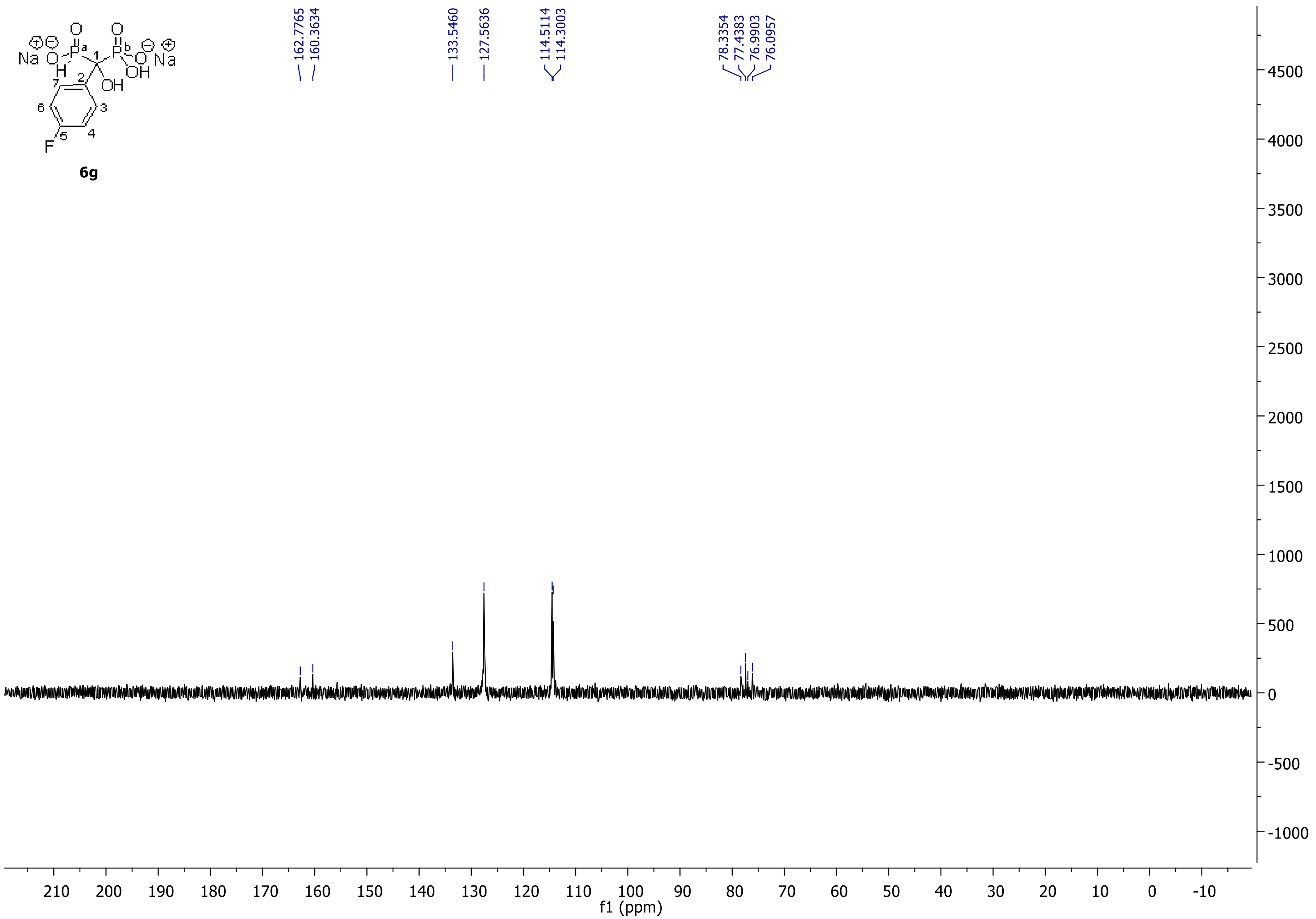

Figure S22: ^{13}C NMR spectrum of of 1-hydroxy-1-(4-fluorophenyl)methane-1,1-bis(*H*-phosphinylphosphonate) disodium salt 6g

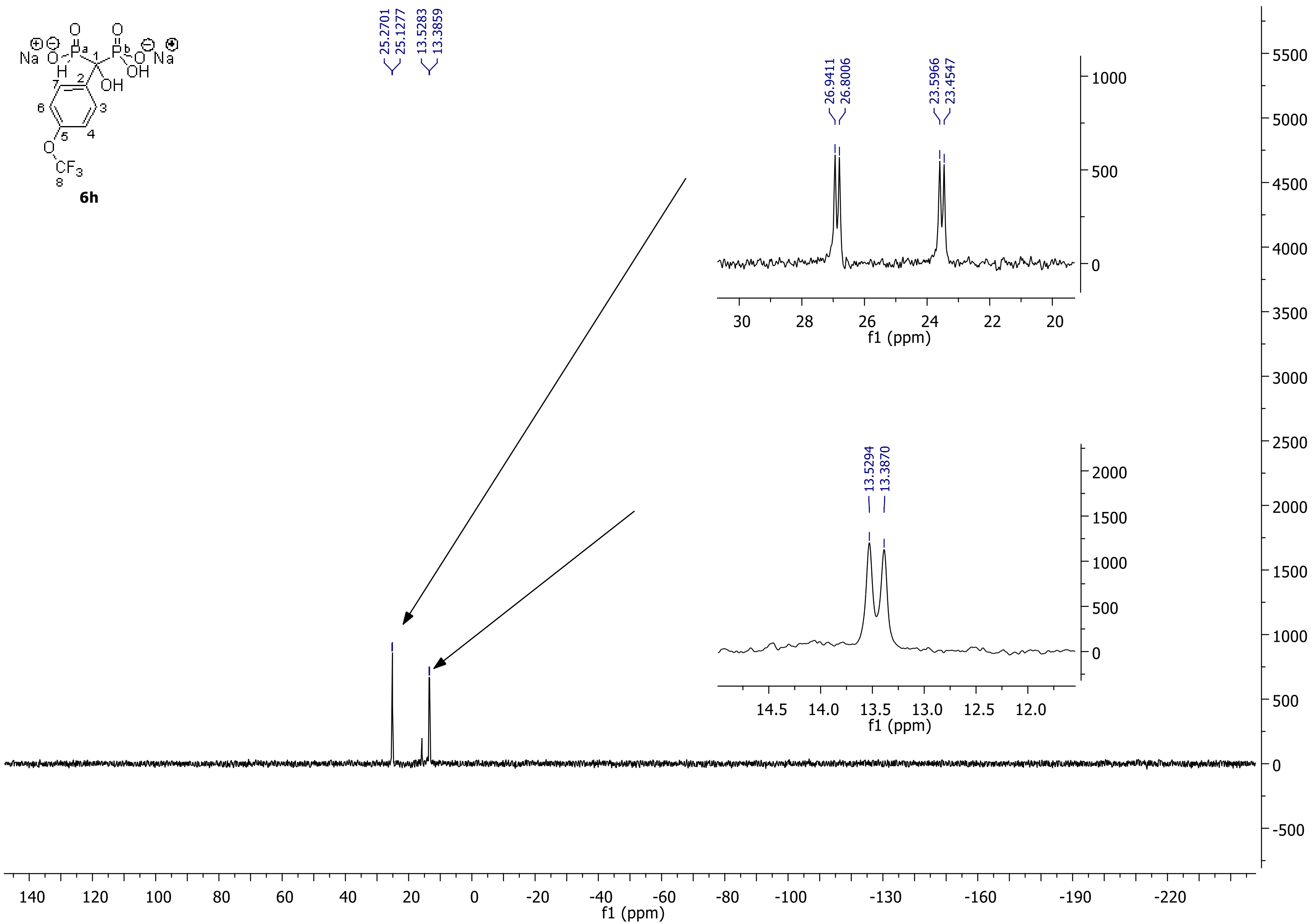

Figure S23: $^{31}P\{^{1}H\}$ and ^{31}P NMR (162 MHz, D_2O) spectra of 1-hydroxy-1-(4-trifluoromethoxyphenyl)methane-1,1-bis(*H*-phosphinylphosphonate) disodium salt 6h

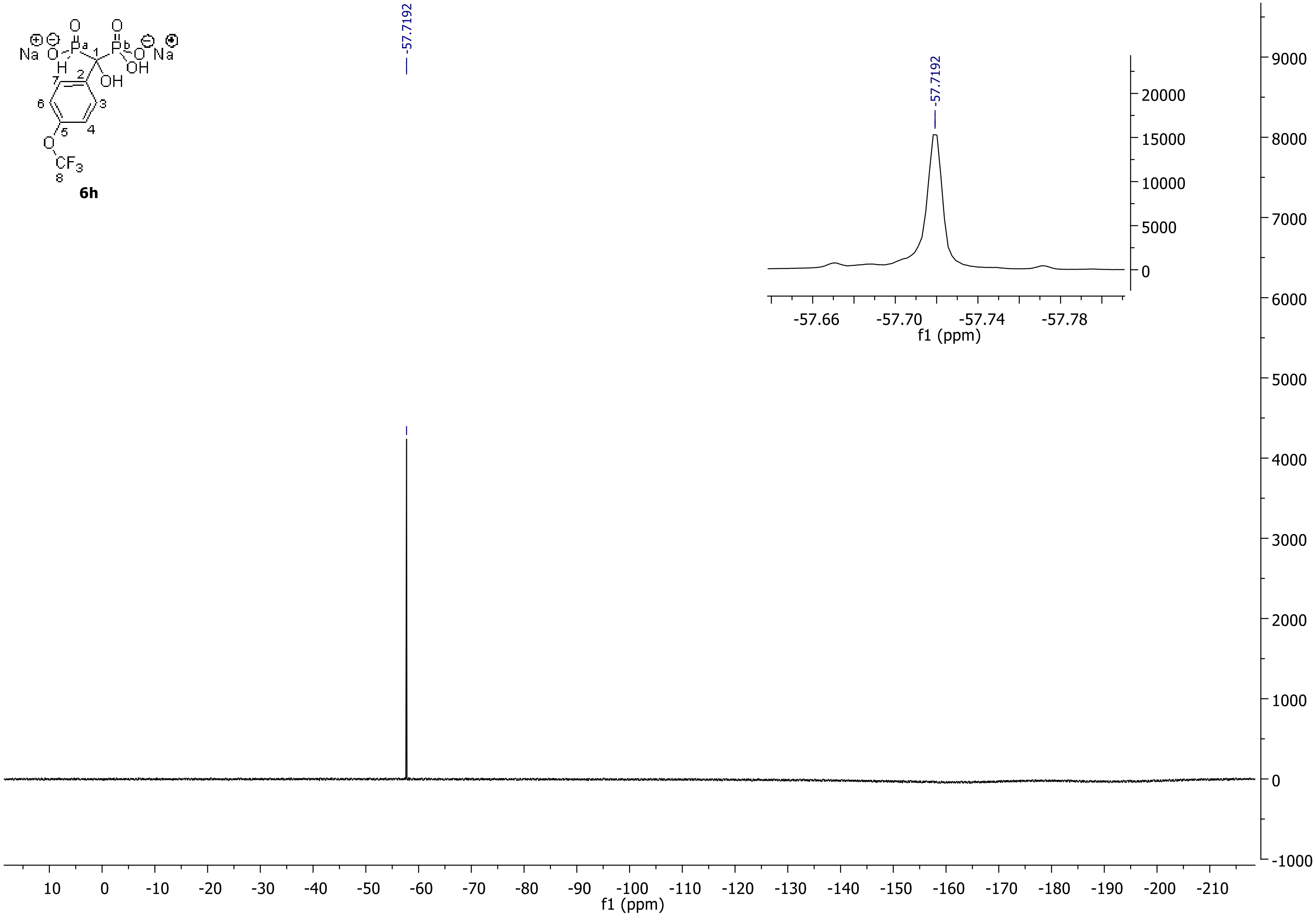

Figure S24: ^{19}F {^{1}H} and ^{19}F (377 MHz, D_2O) spectra of 1-hydroxy-1-(4-trifluoromethoxyphenyl)methane-1,1-bis(*H*-phosphinylphosphonate) disodium salt 6h

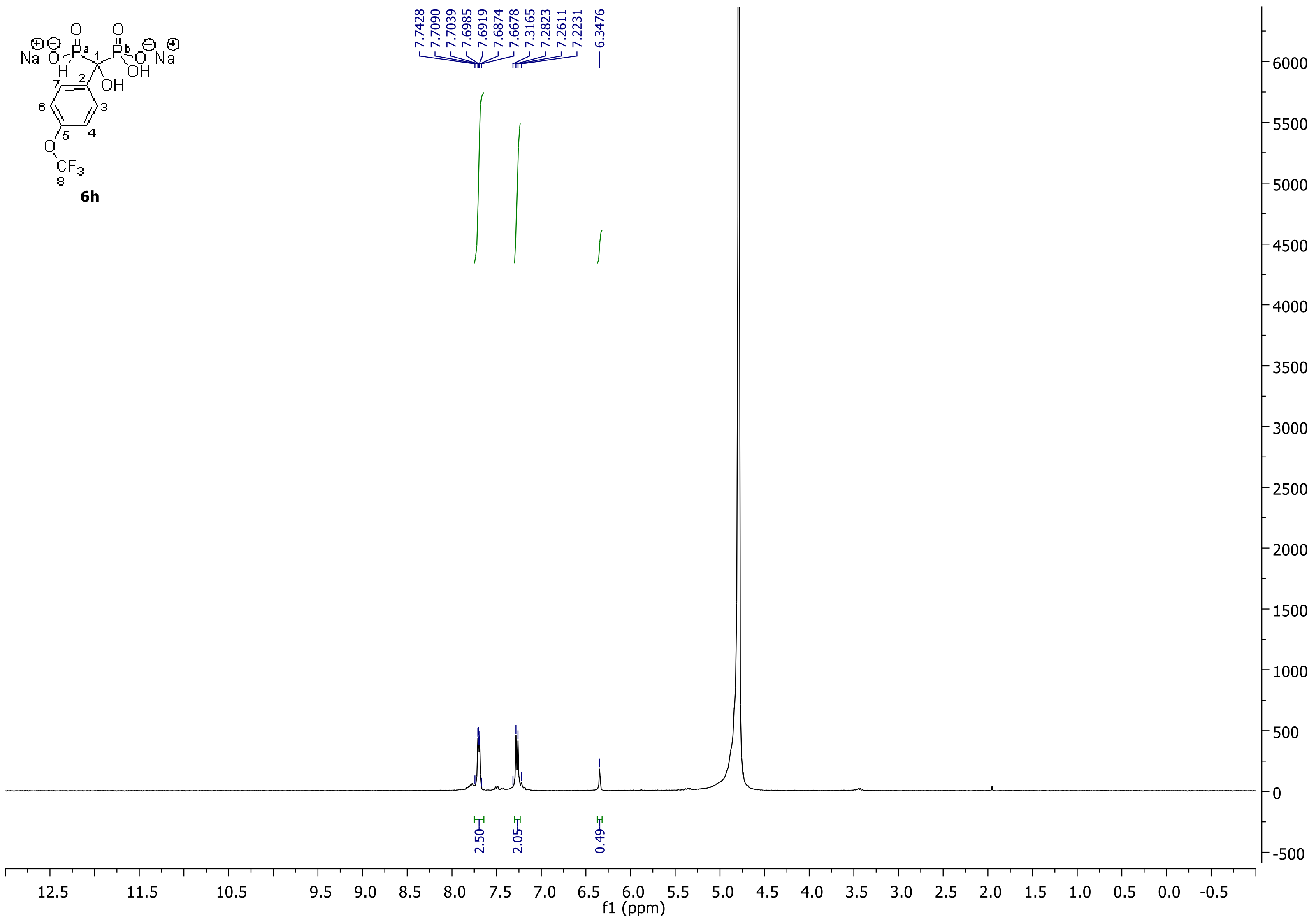

Figure S25: ^{1}H NMR spectrum (400 MHz, D_2O) of 1-hydroxy-1-(4-trifluoromethoxyphenyl)methane-1,1-bis(*H*-phosphinylphosphonate) disodium salt 6h

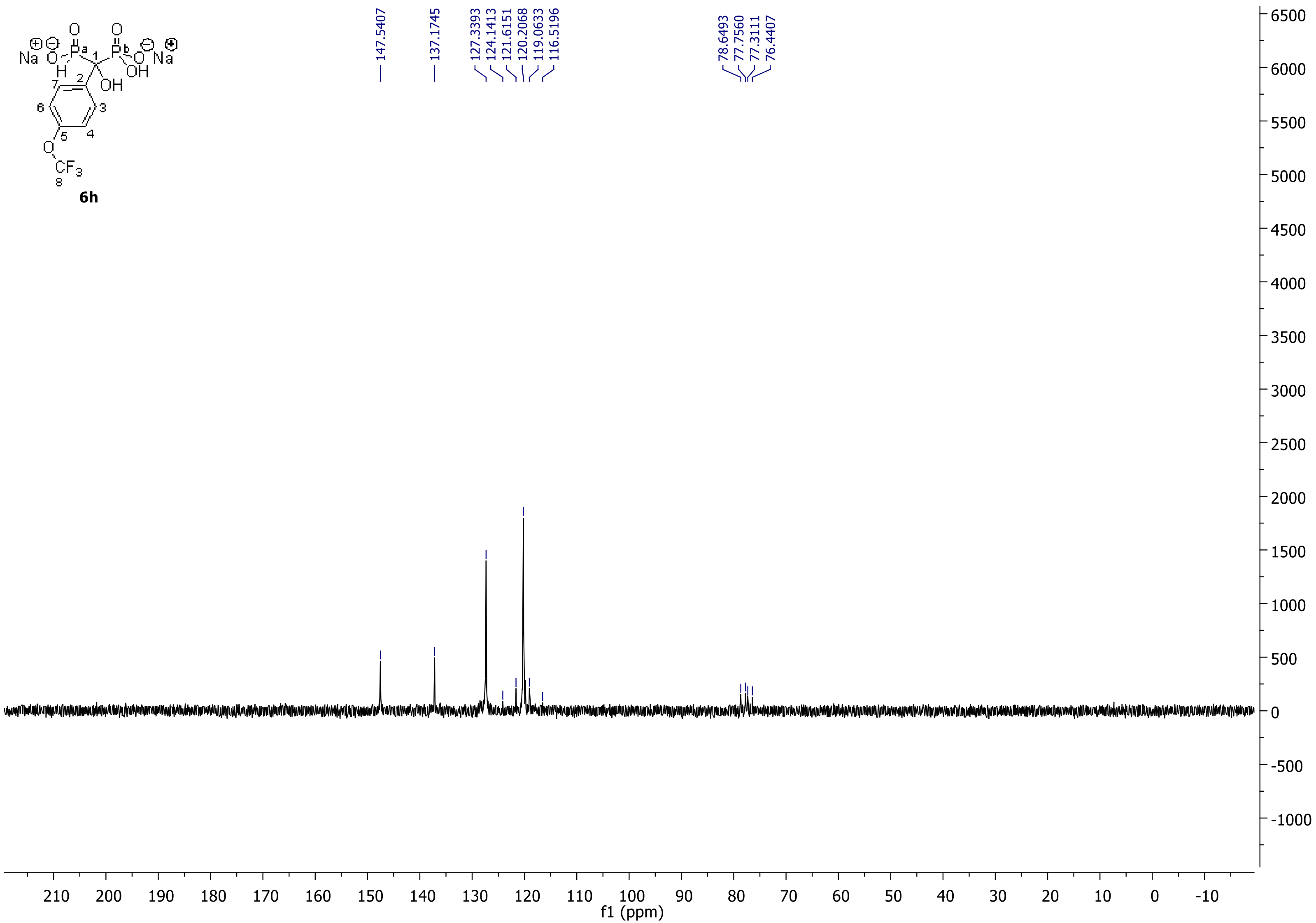

Figure S26: ^{13}C NMR (101 MHz, D_2O) spectrum of 1-hydroxy-1-(4-trifluoromethoxyphenyl)methane-1,1-bis(*H*-phosphinylphosphonate) disodium salt 6h

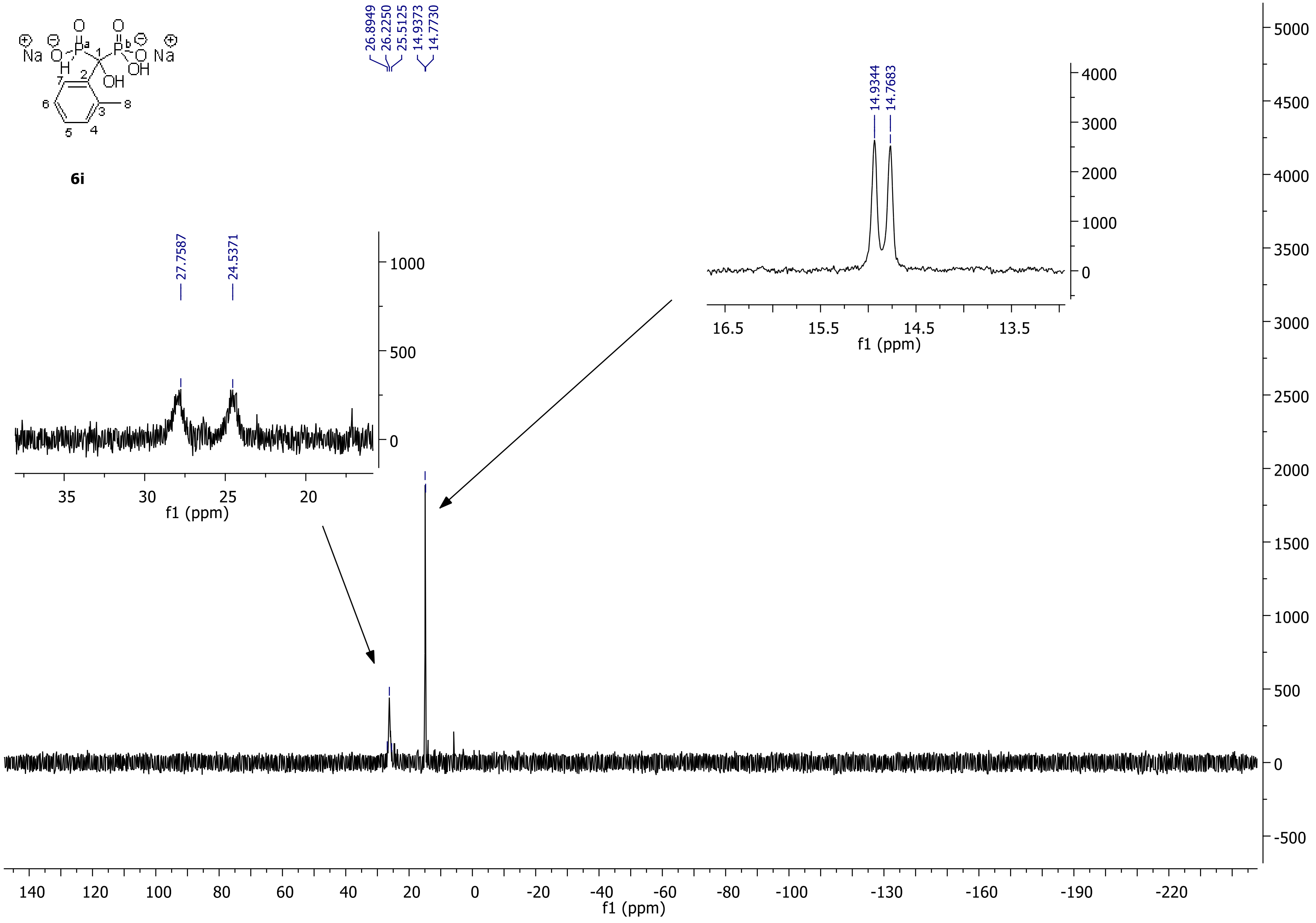

Figure S27: $^{31}P\{^{1}H\}$ and ^{31}P NMR (162 MHz, D_2O) spectra of 1-hydroxy-1-(2-tolyl)methane-1,1-bis(*H*-phosphonate) disodium salt 6i

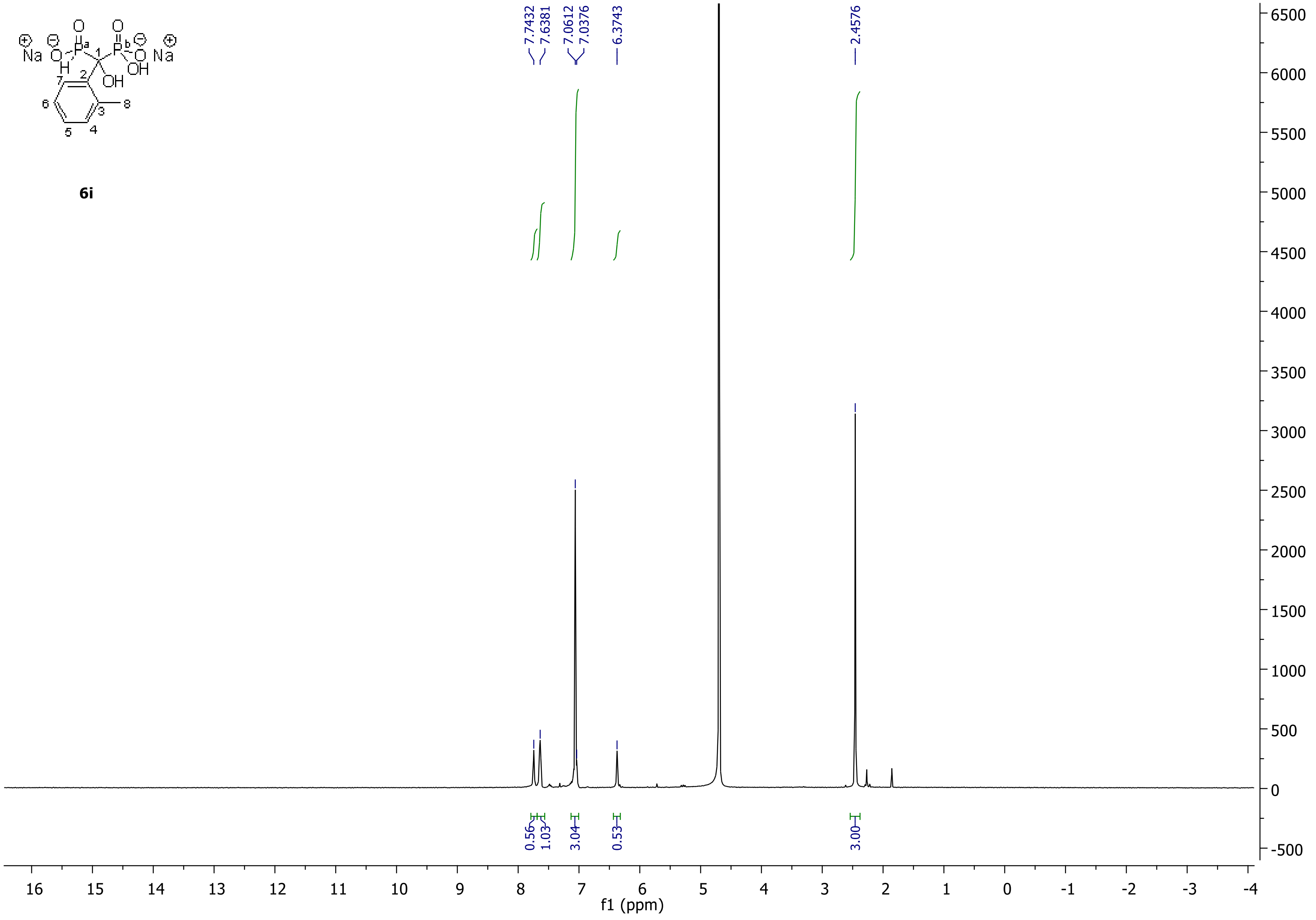

Figure S28: ^{1}H NMR (400 MHz, D_2O) spectrum of 1-hydroxy-1-(2-tolyl)methane-1,1-bis(*H*-phosphonate) disodium salt 6i

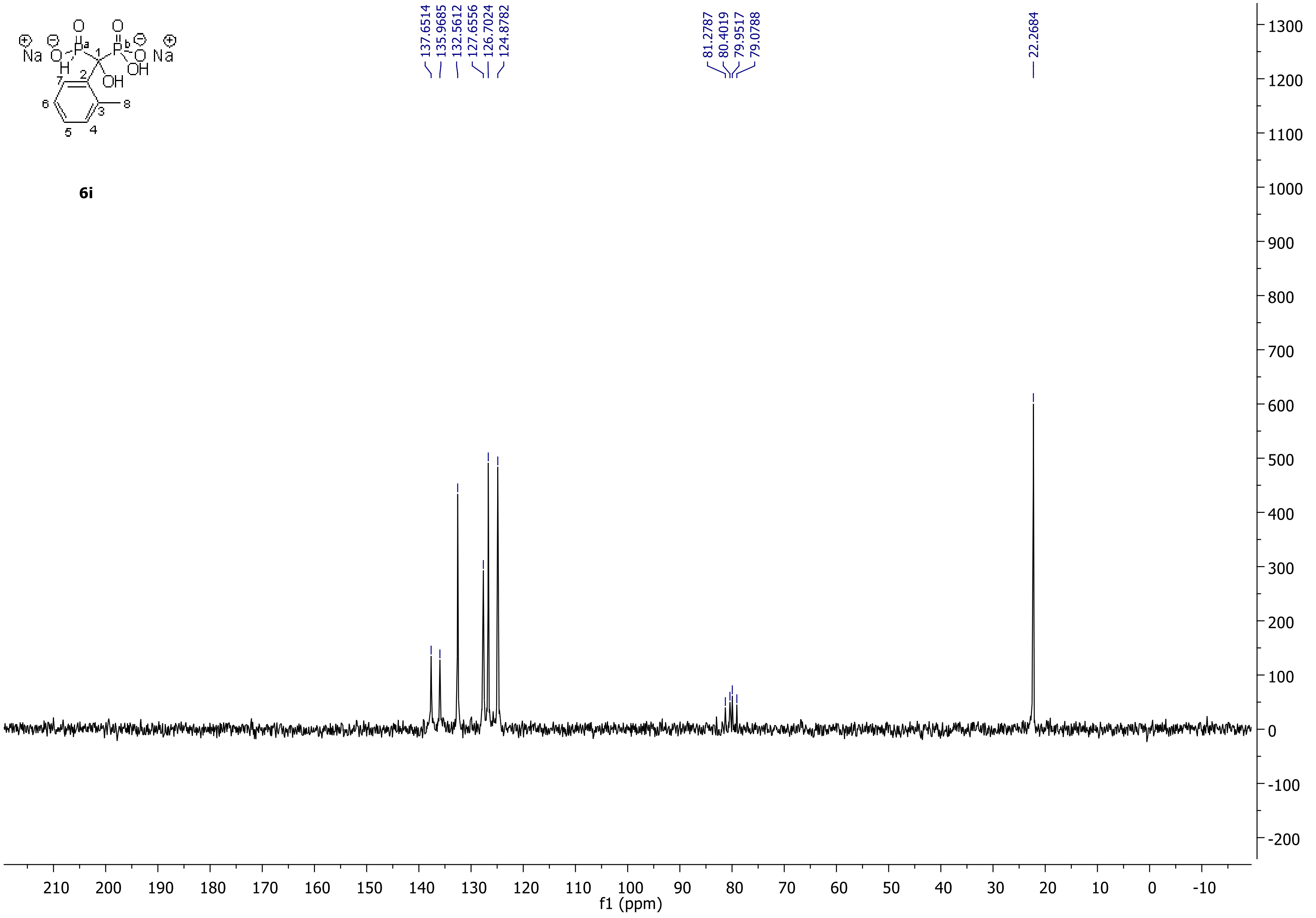

Figure S29: ^{13}C NMR (101 MHz, D_2O) spectrum of 1-hydroxy-1-(2-tolyl)methane-1,1-bis(*H*-phosphonate) disodium salt 6i

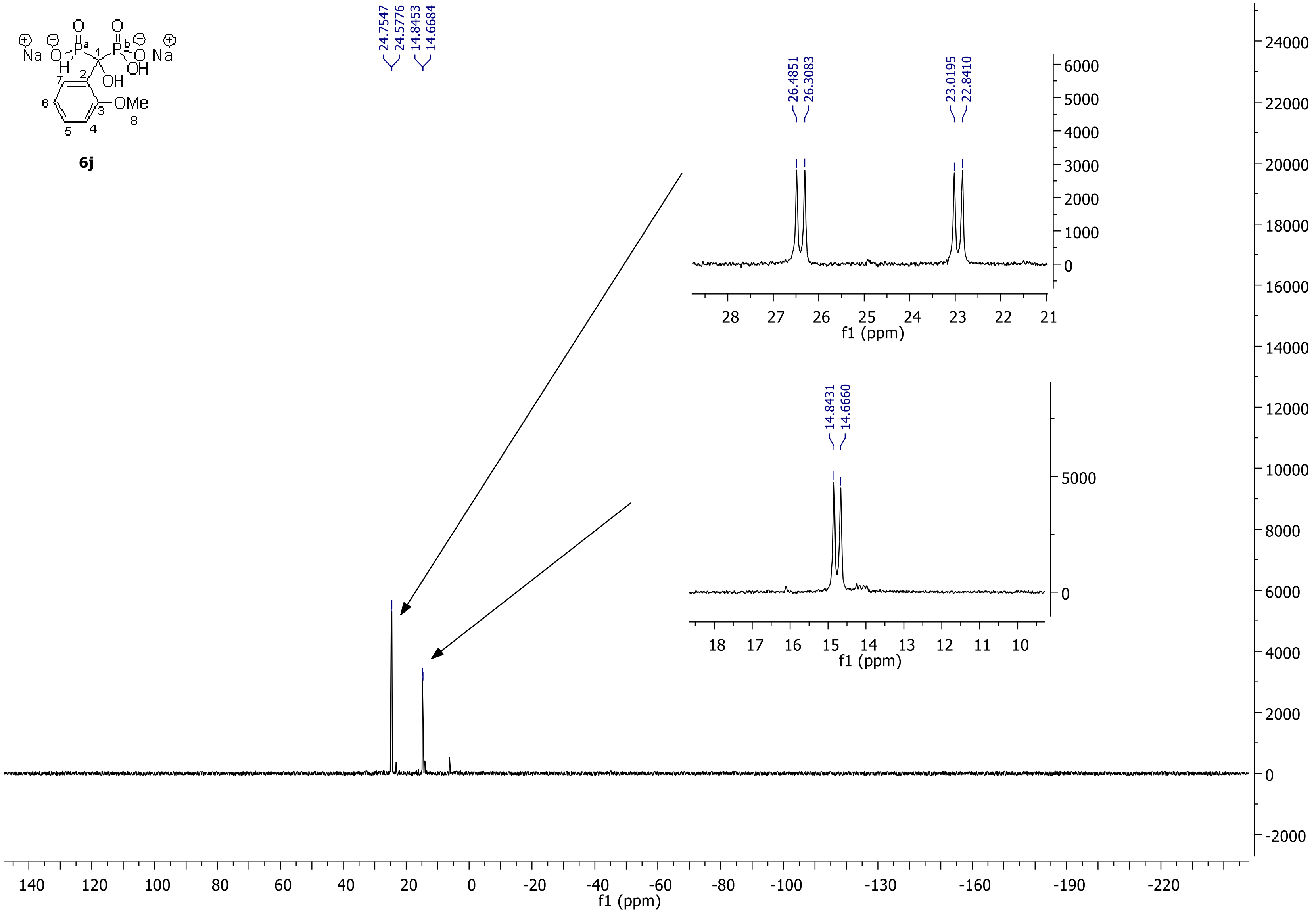

Figure S30: $^{31}P\{^{1}H\}$ and ^{31}P NMR (162 MHz, D_2O) spectra of 1-hydroxy-1-(2-methoxyphenyl)methane-1,1-bis(*H*-phosphinylphosphonate) disodium salt 6j

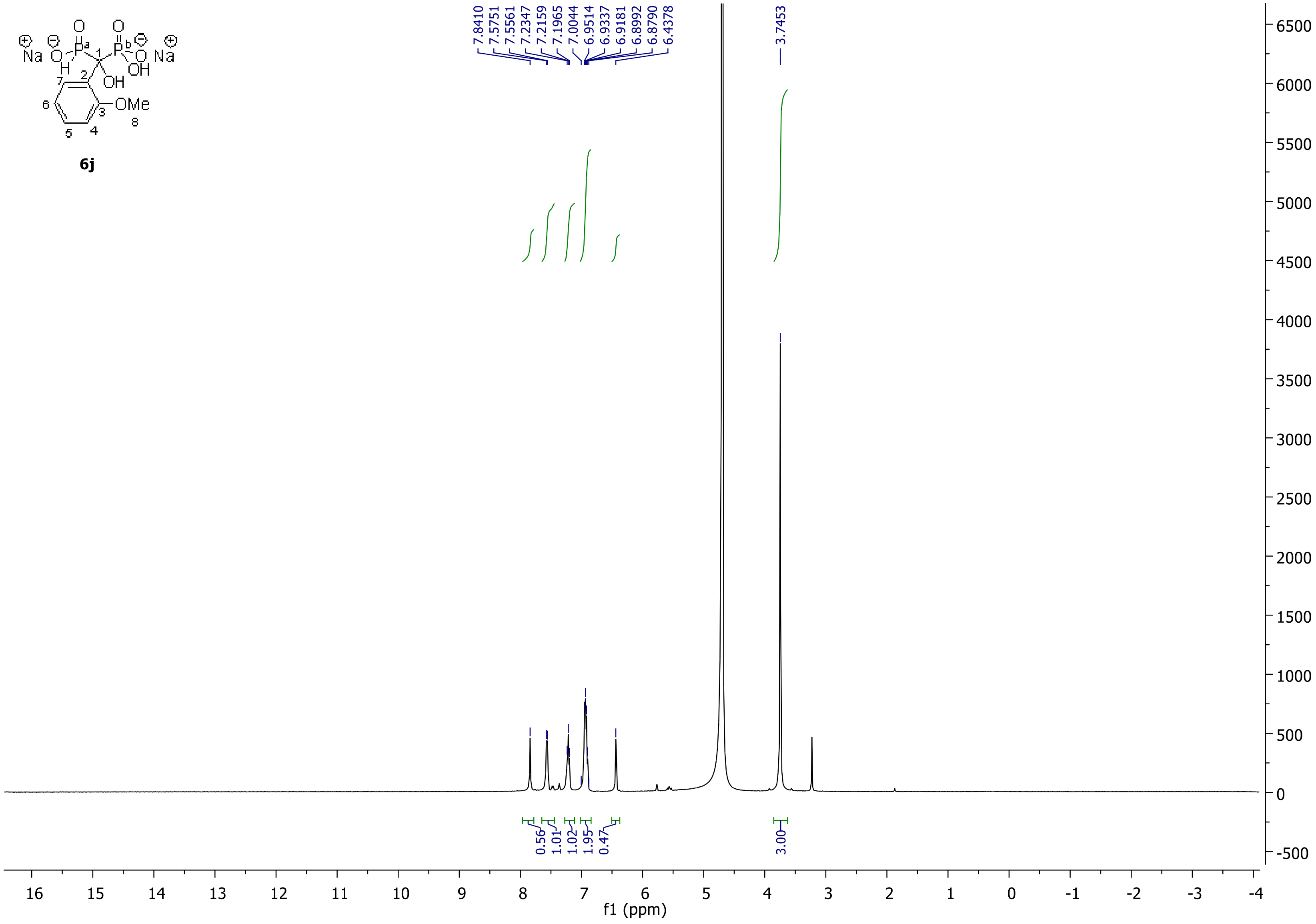

Figure S31: ^{1}H NMR (400 MHz, D_2O) spectrum of 1-hydroxy-1-(2-methoxyphenyl)methane-1,1-bis(*H*-phosphinylphosphonate) disodium salt 6j

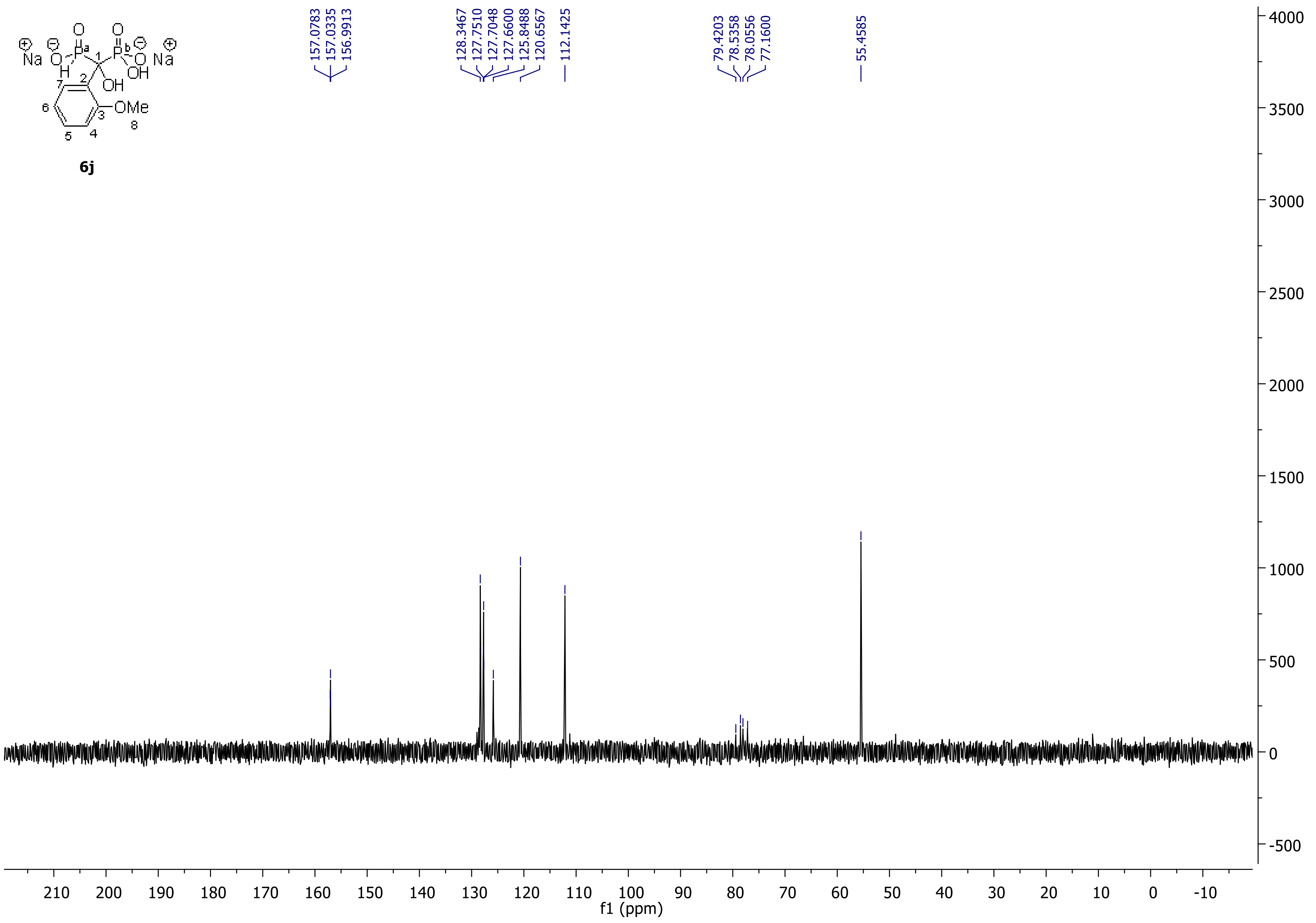

Figure S32: ^{13}C NMR spectrum (101 MHz, D_2O) of 1-hydroxy-1-(2-methoxyphenyl)methane-1,1-bis(*H*-phosphinylphosphonate) disodium salt 6j

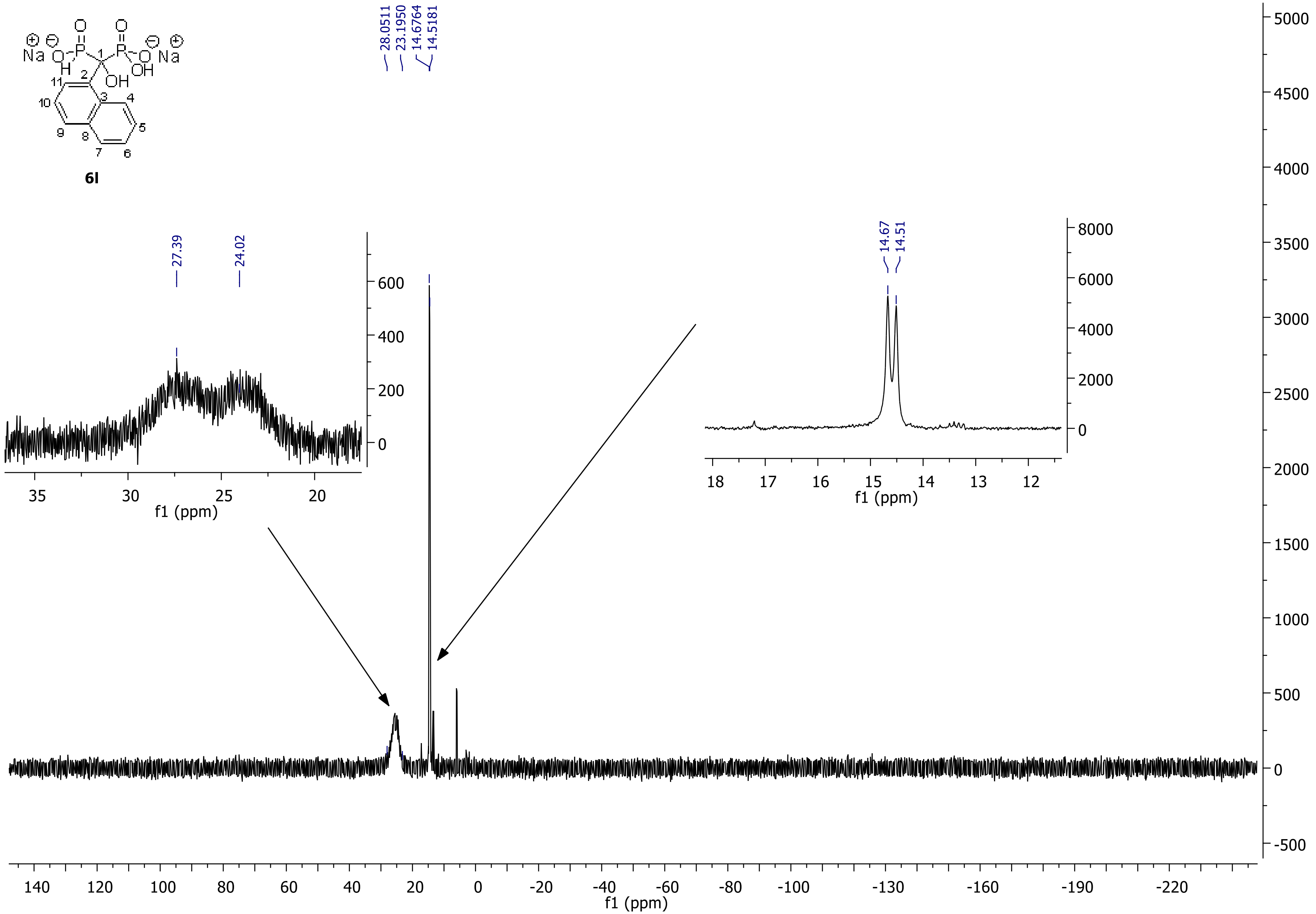

Figure S33: $^{31}P\{^{1}H\}$ and ^{31}P NMR (162 MHz, D_2O) spectra of 1-hydroxy-1-(1-napthyl)methane-1,1-bis(*H*-phosphinylphosphonate) disodium salt 6l

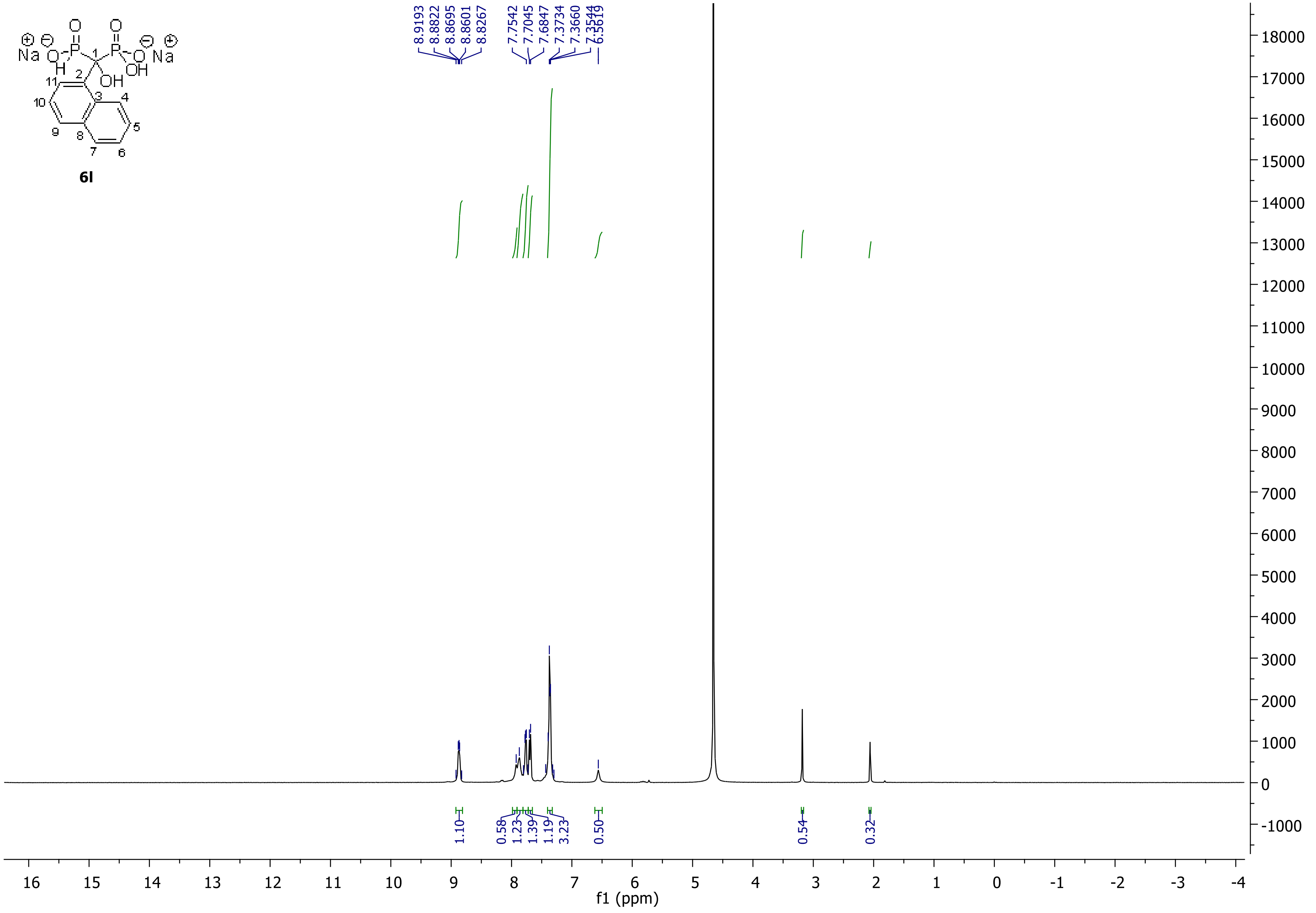

Figure S34: ^{1}H NMR (400 MHz, D_2O) spectrum of 1-hydroxy-1-(1-napthyl)methane-1,1-bis(*H*-phosphinylphosphonate) disodium salt 6l

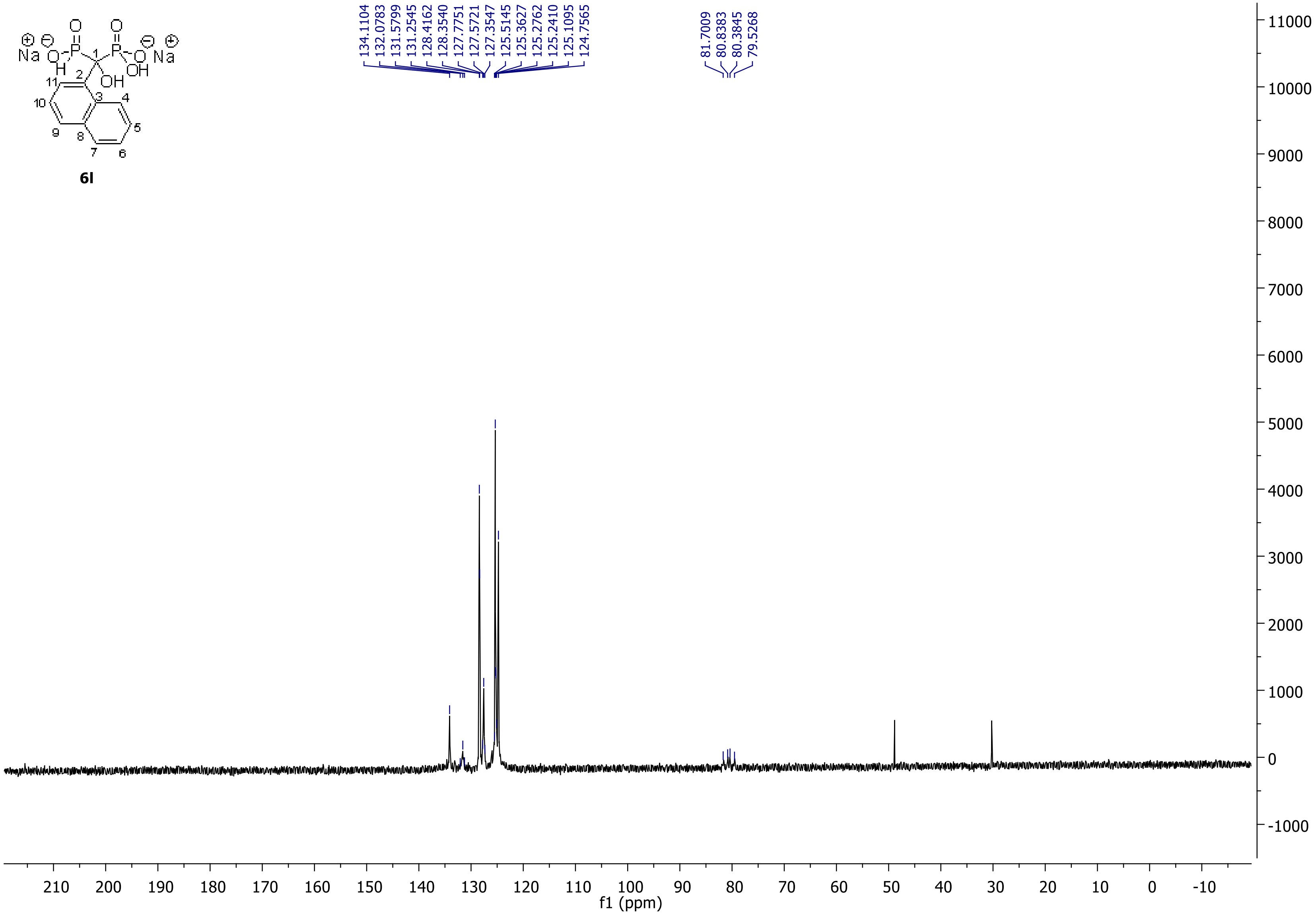

Figure S35: ^{13}C NMR spectrum (101 MHz, D_2O) of 1-hydroxy-1-(1-napthyl)methane-1,1-bis(*H*-phosphinylphosphonate) disodium salt 6l

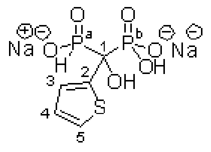

6m

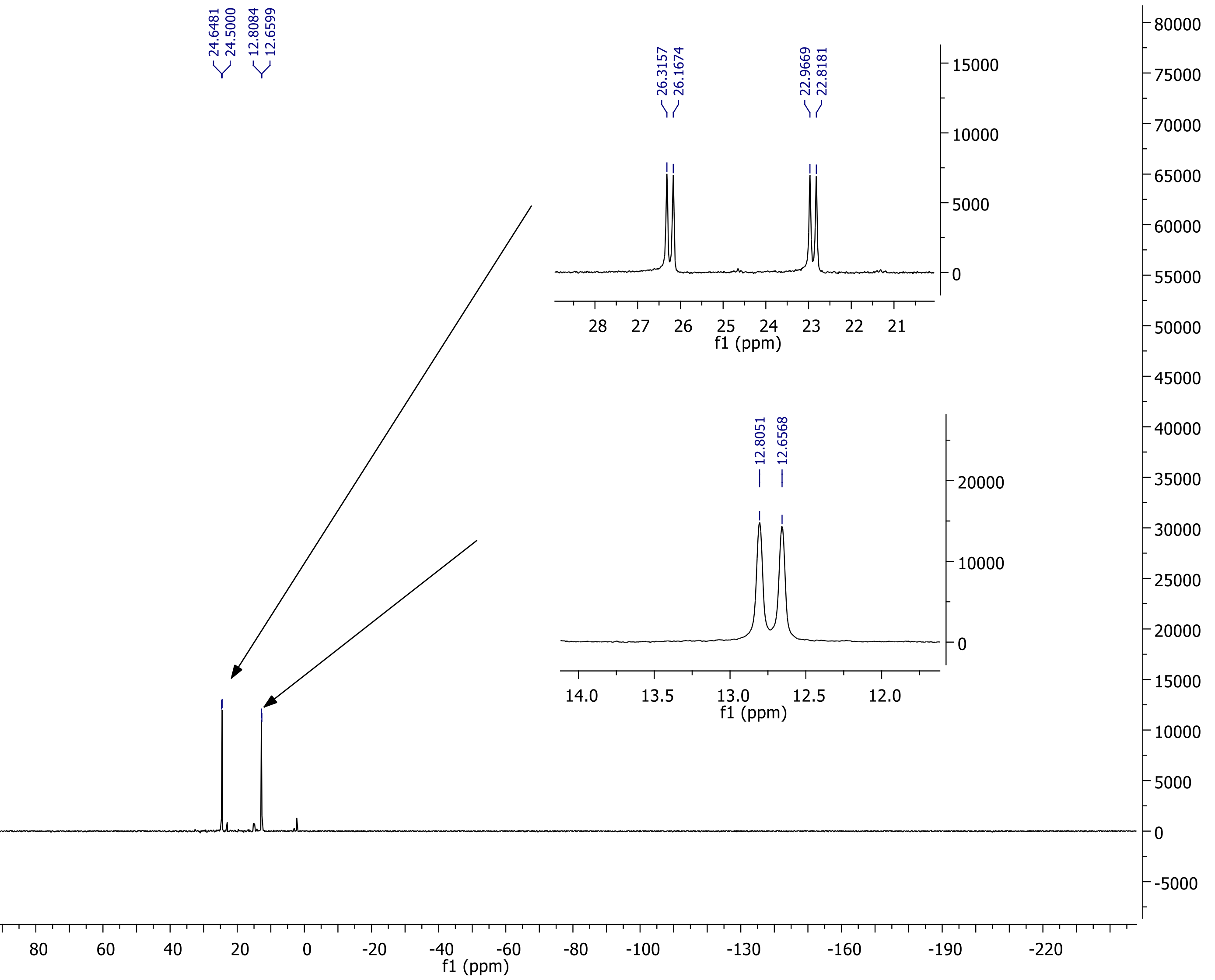

Figure S36: $^{31}P\{^{1}H\}$ and ^{31}P NMR (162 MHz, D_2O) spectra of 1-hydroxy-1-(2-thienyl)methane-1,1-bis(*H*-phosphinylphosphonate) disodium salt 6m

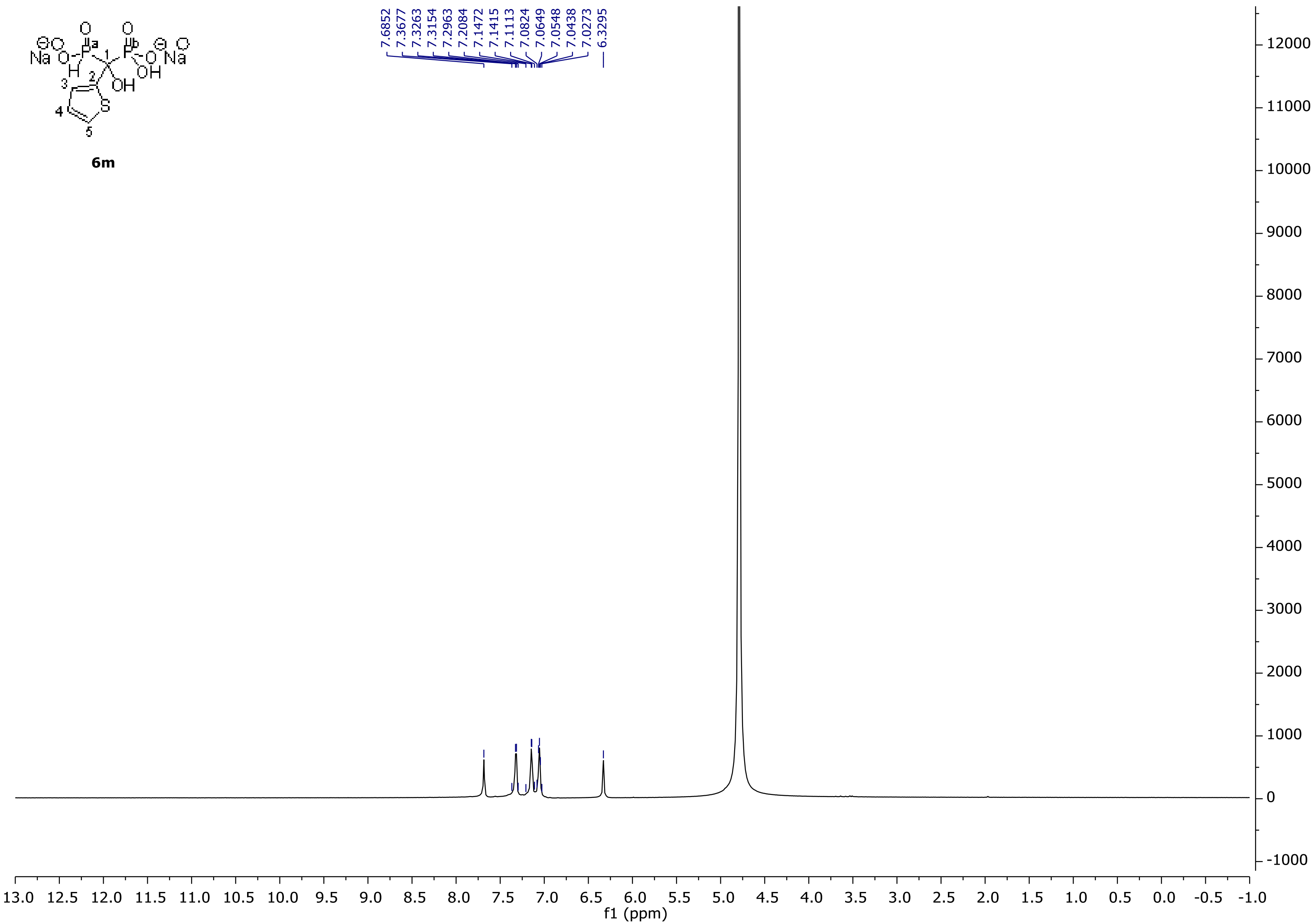

Figure S37: ^{1}H NMR (400 MHz, D_2O) spectrum of 1-hydroxy-1-(2-thienyl)methane-1,1-bis(*H*-phosphinylphosphonate) disodium salt 6m

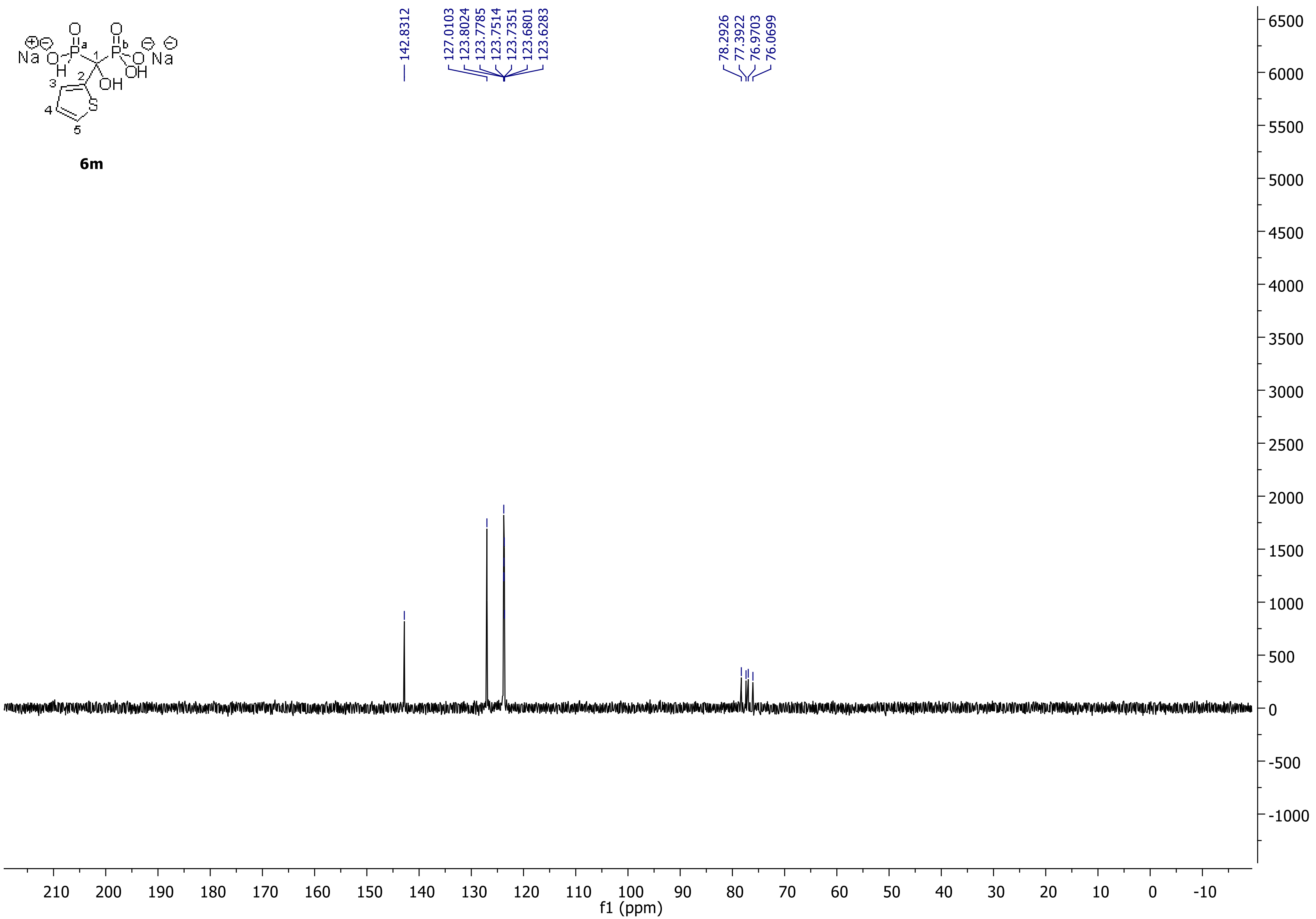

Figure S38: ^{13}C NMR (101 MHz, D_2O) spectrum of 1-hydroxy-1-(2-thienyl)methane-1,1-bis(*H*-phosphinylphosphonate) disodium salt 6m

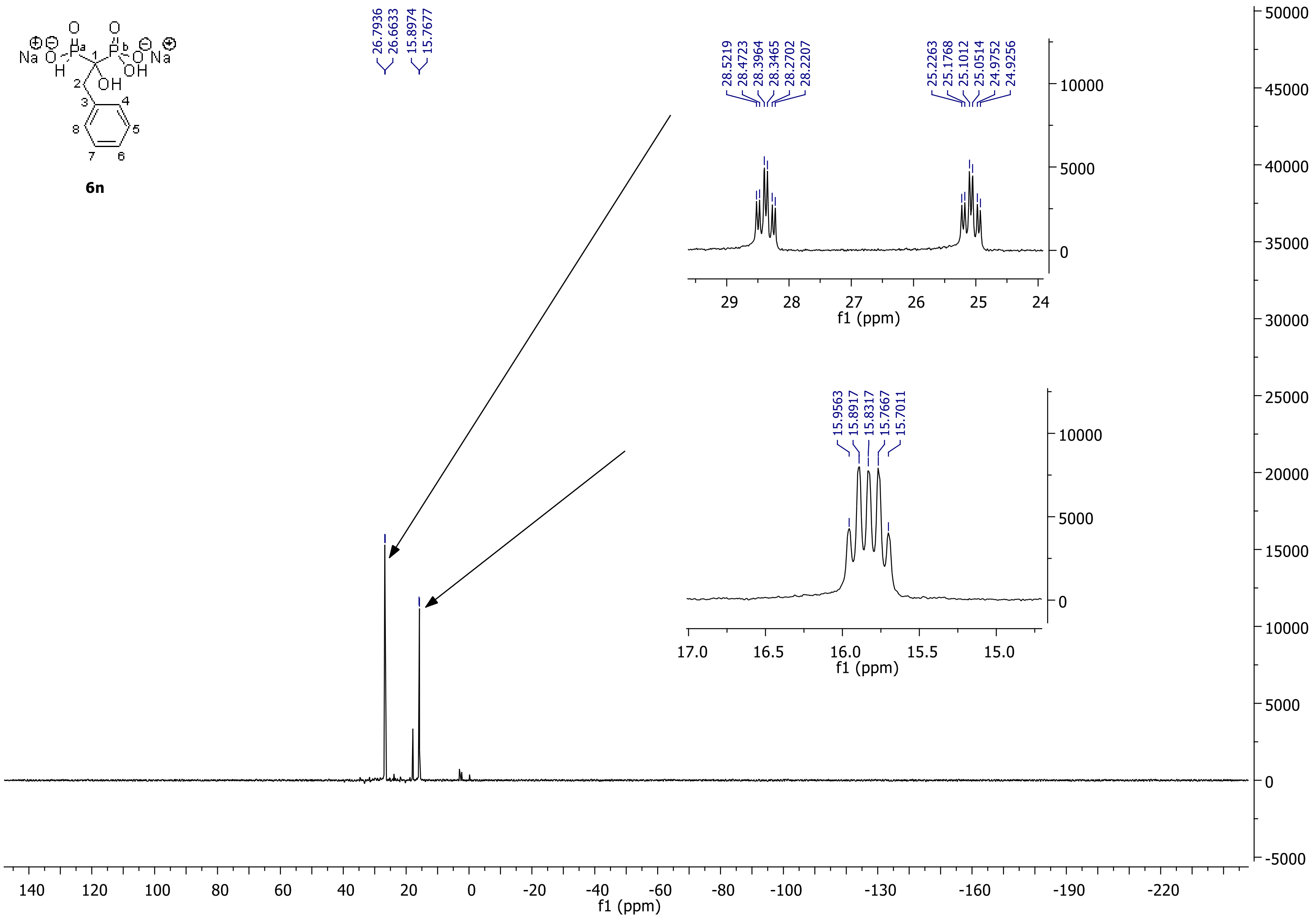

Figure S39: $^{31}P\{^1H\}$ and ^{31}P NMR (162 MHz, D_2O) spectra of 1-hydroxy-2-phenylethane-1,1-bis(*H*-phosphinylphosphonate) disodium salt 6n

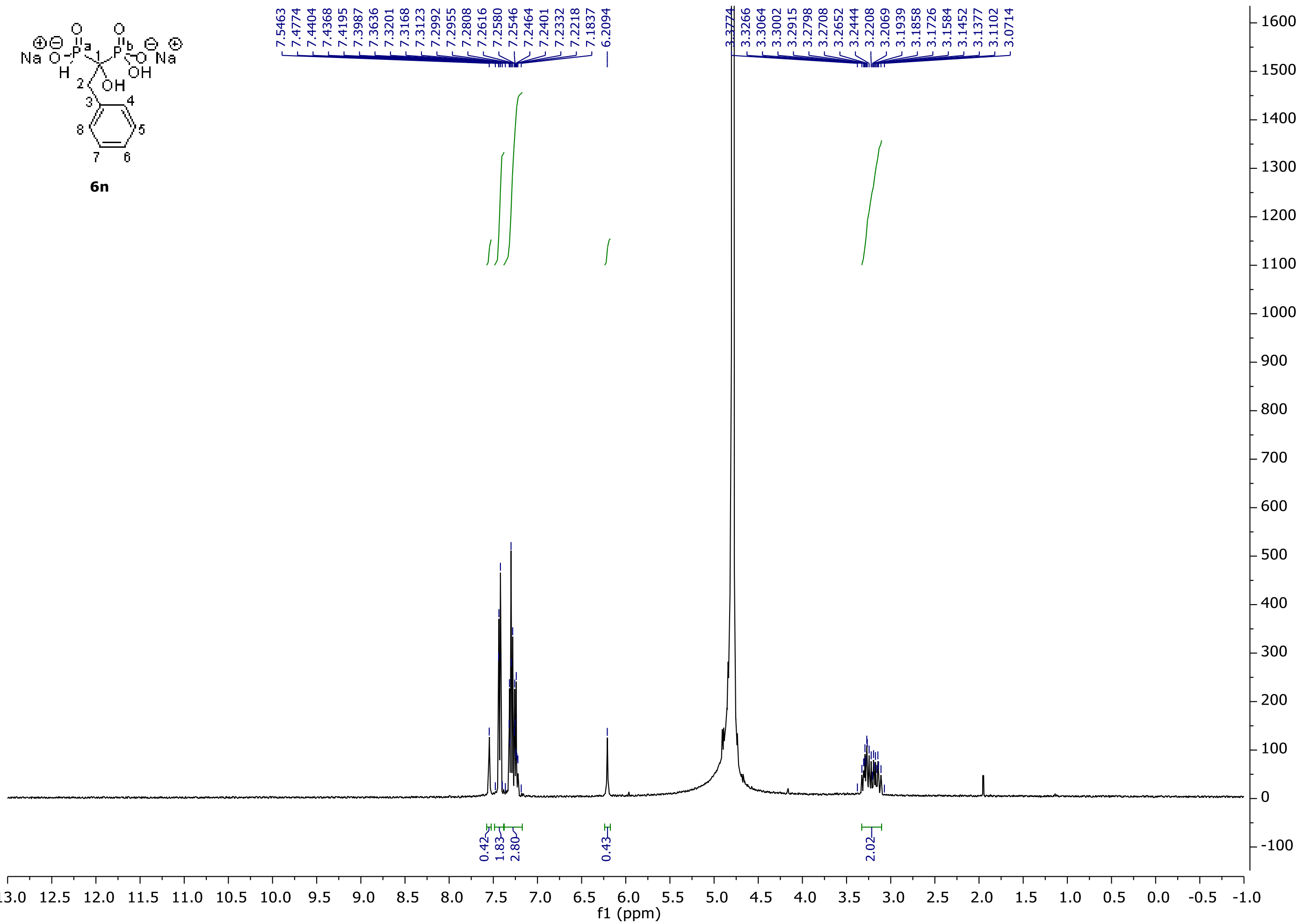

Figure S40: ^{1}H NMR (400 MHz, D_2O) spectrum of 1-hydroxy-2-phenylethane-1,1-bis(*H*-phosphinylphosphonate) disodium salt 6n

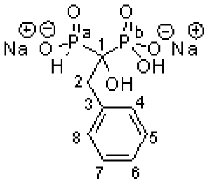

6n

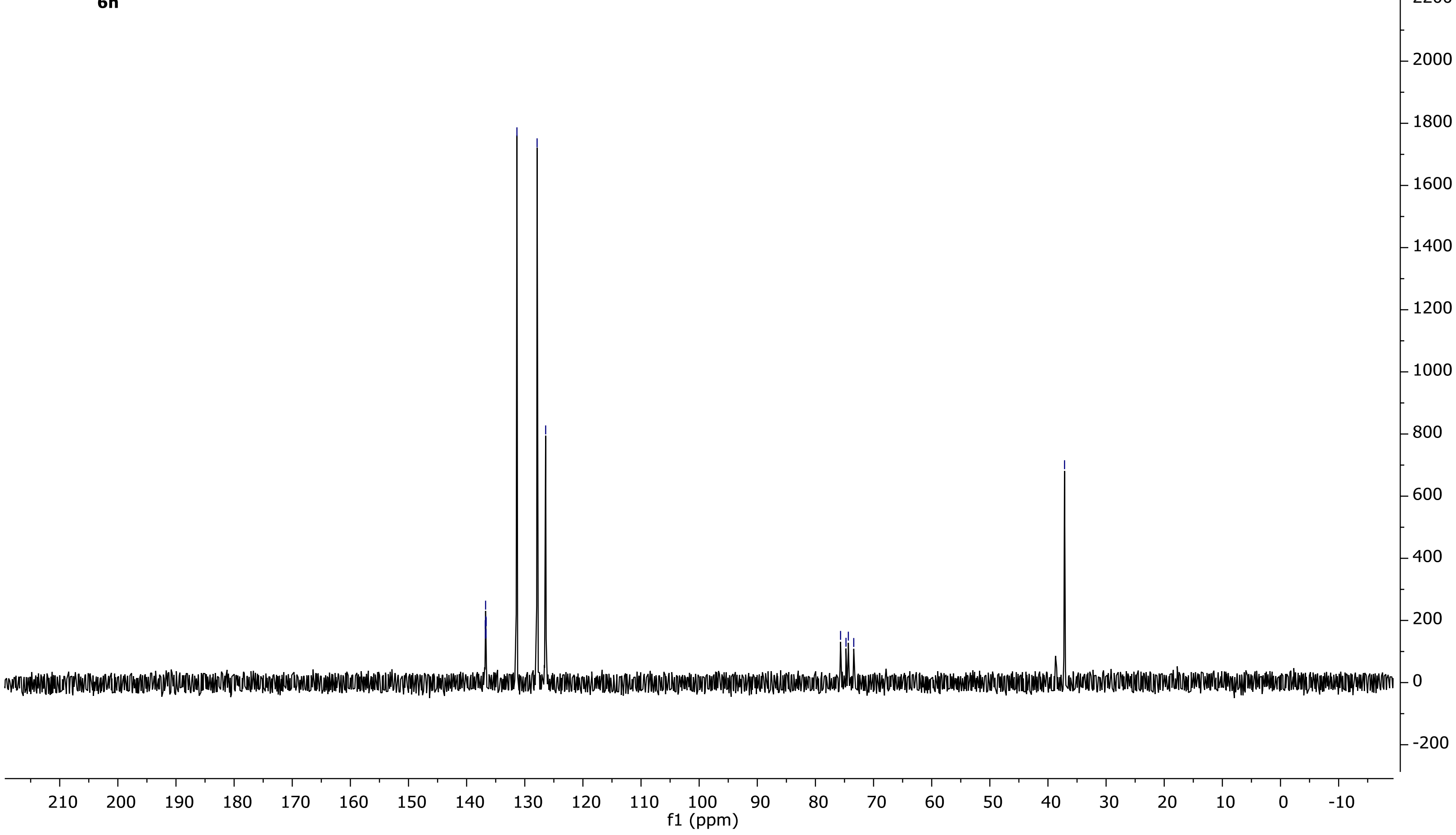

Figure S41: ^{13}C NMR (101 MHz, D_2O) spectrum of 1-hydroxy-2-phenylethane-1,1-bis(*H*-phosphinylphosphonate) disodium salt 6n

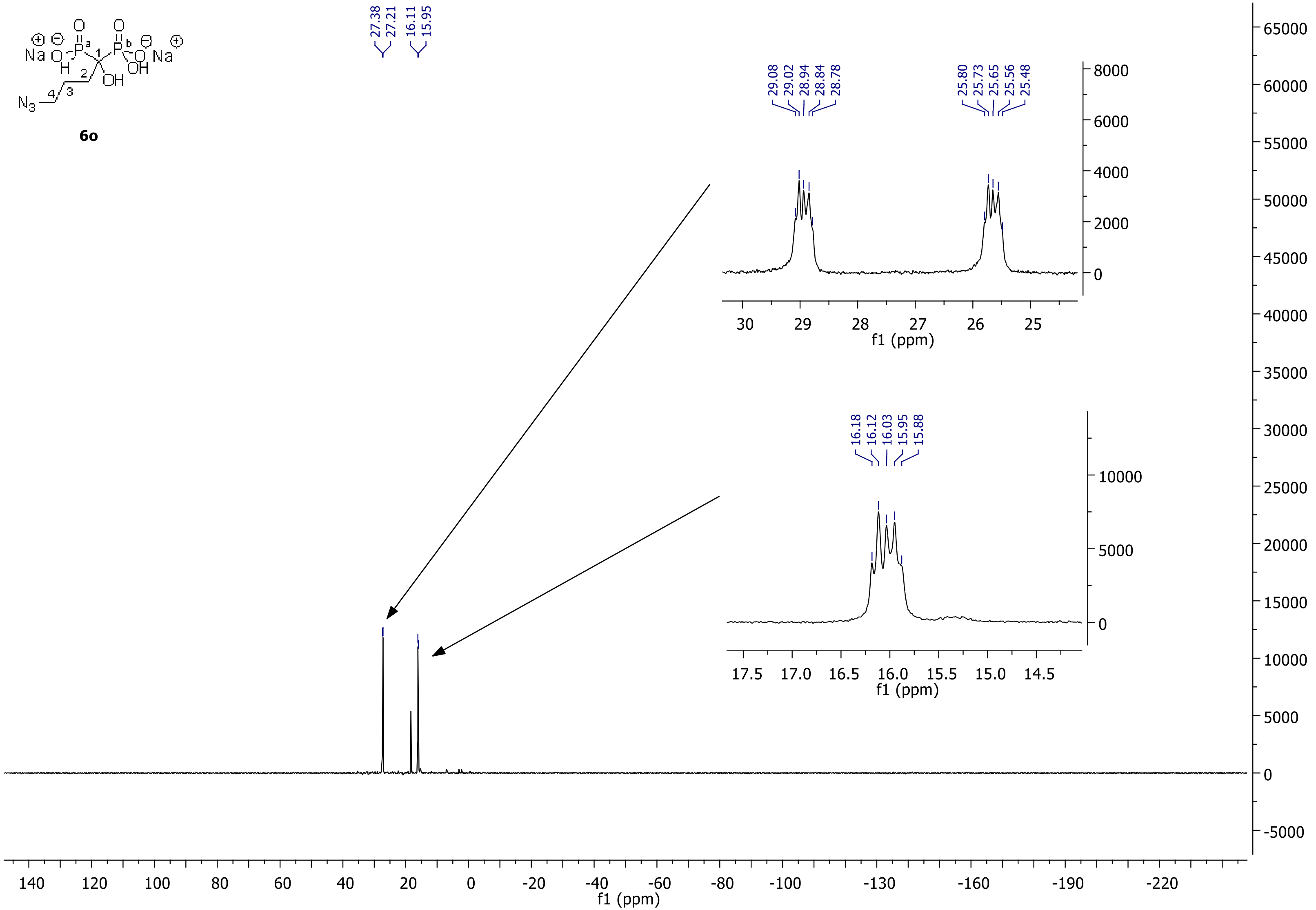

Figure S42: $^{31}P\{^1H\}$ and ^{31}P NMR (162 MHz, D_2O) spectra of 1-hydroxy-1-(3-azidopropyl)methane-1,1-bis(*H*-phosphinylphosphonate) disodium salt 6o

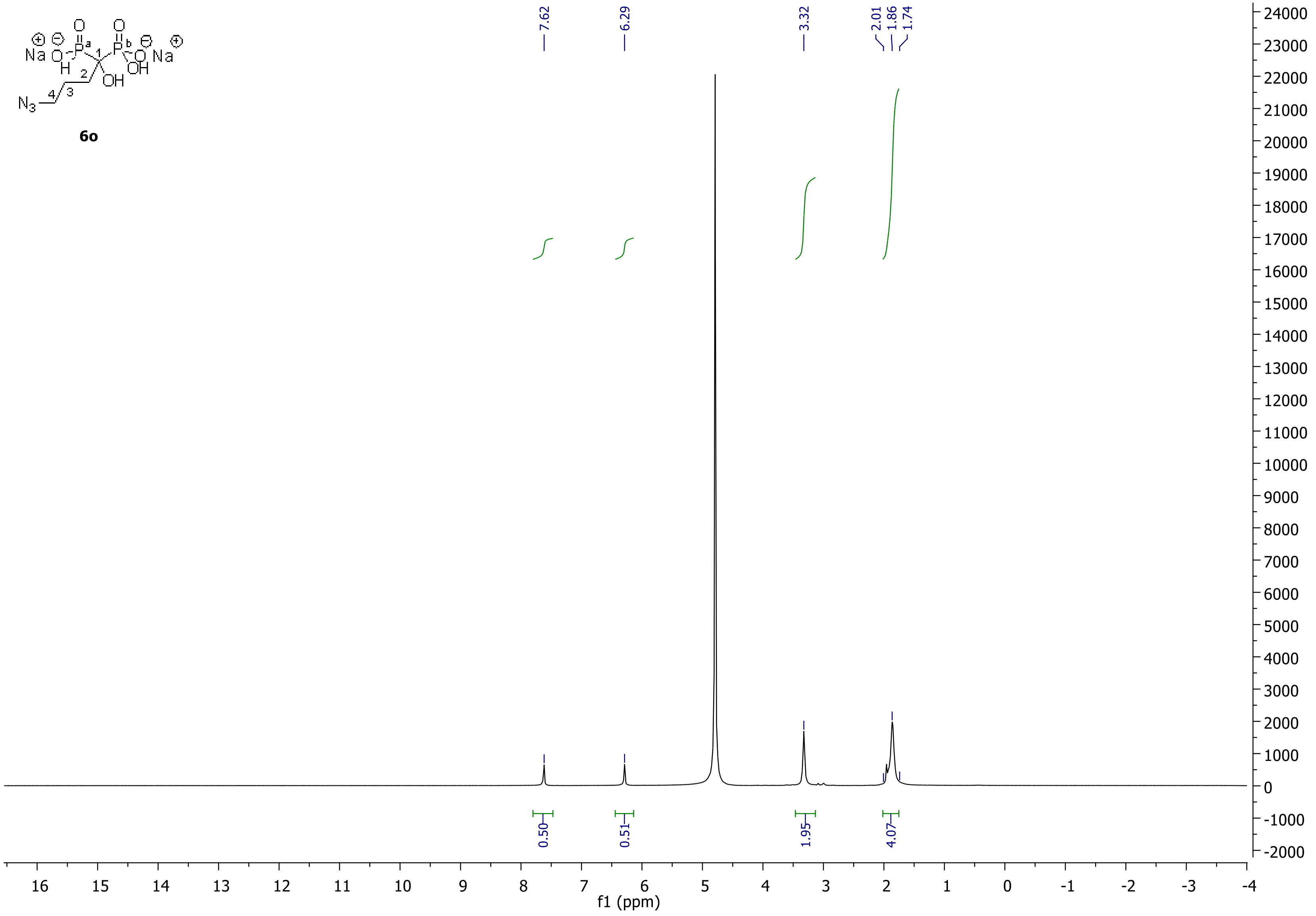

Figure S43: ^{1}H NMR (400 MHz, D_2O) spectrum of 1-hydroxy-1-(3-azidopropyl)methane-1,1-bis(*H*-phosphinylphosphonate) disodium salt 6o

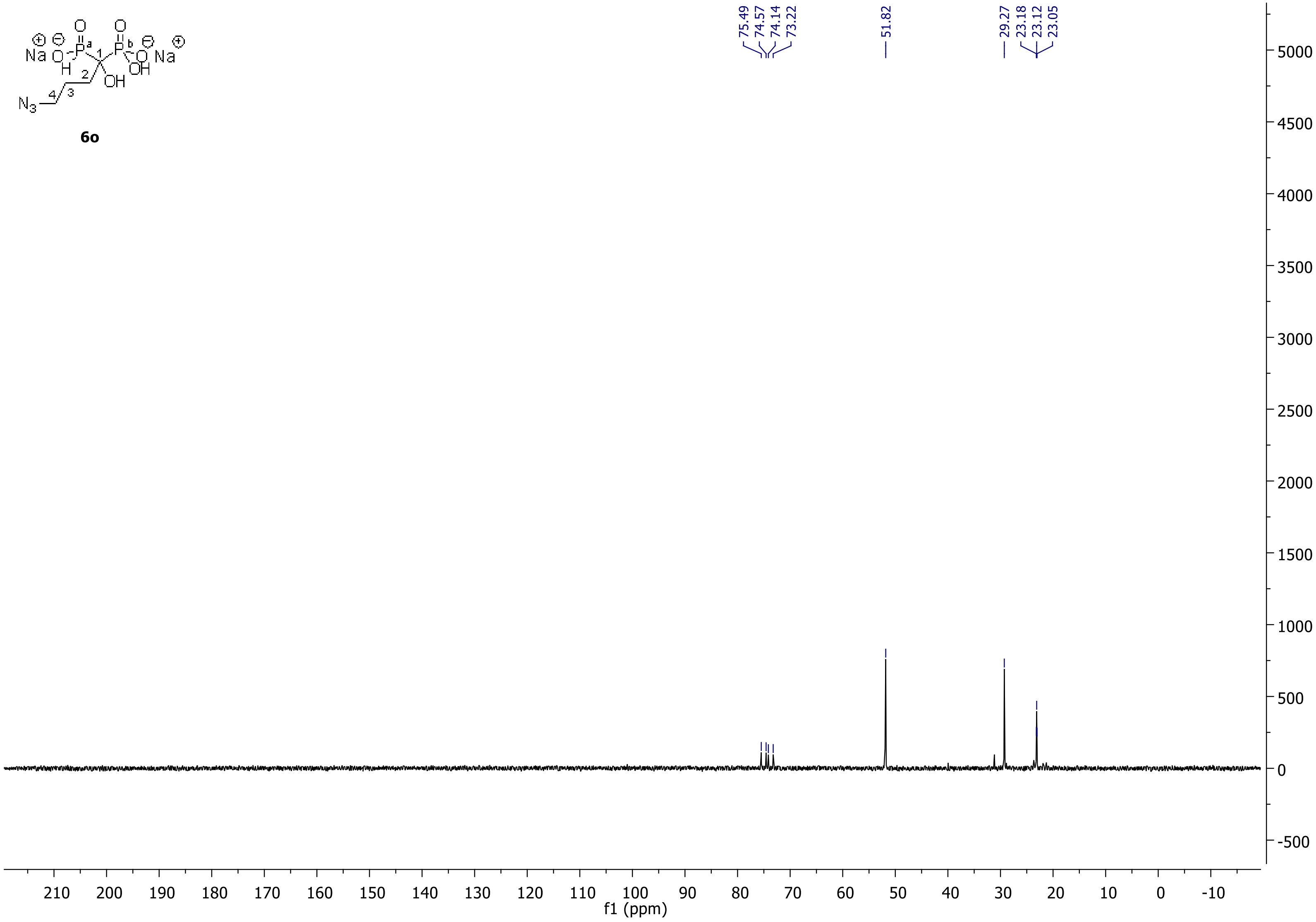

Figure S44: ^{13}C NMR (101 MHz, D_2O) spectrum of 1-hydroxy-1-(3-azidopropyl)methane-1,1-bis(*H*-phosphinylphosphonate) disodium salt 6o

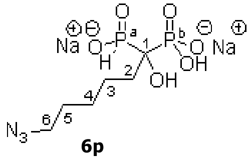

6p

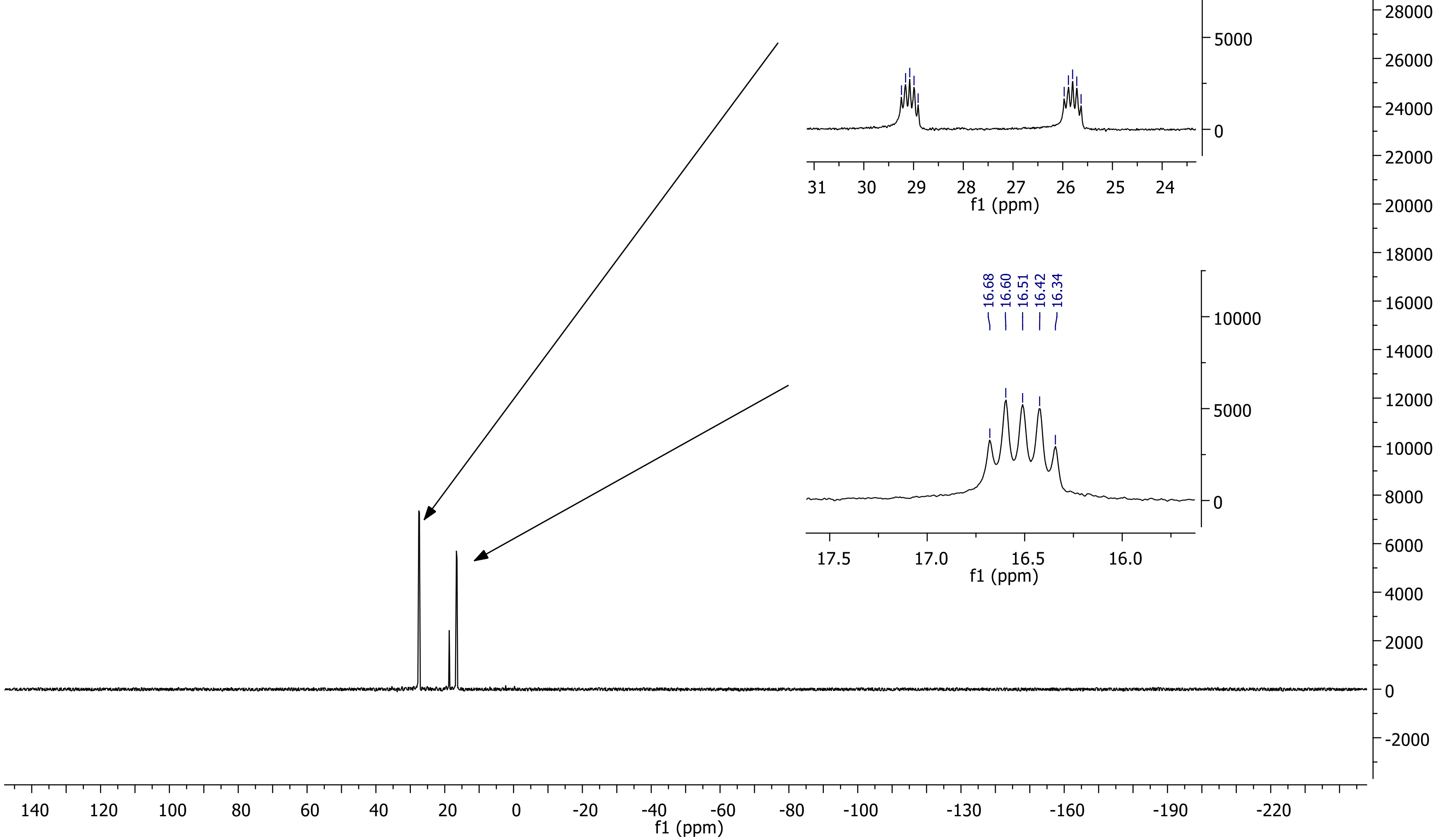

Figure S45: $^{31}P\{^{1}H\}$ and ^{31}P NMR (162 MHz, D_2O) spectra of 1-hydroxy-1-(5-azidopentyl)methane-1,1-bis(*H*-phosphinylphosphonate) disodium salt 6p

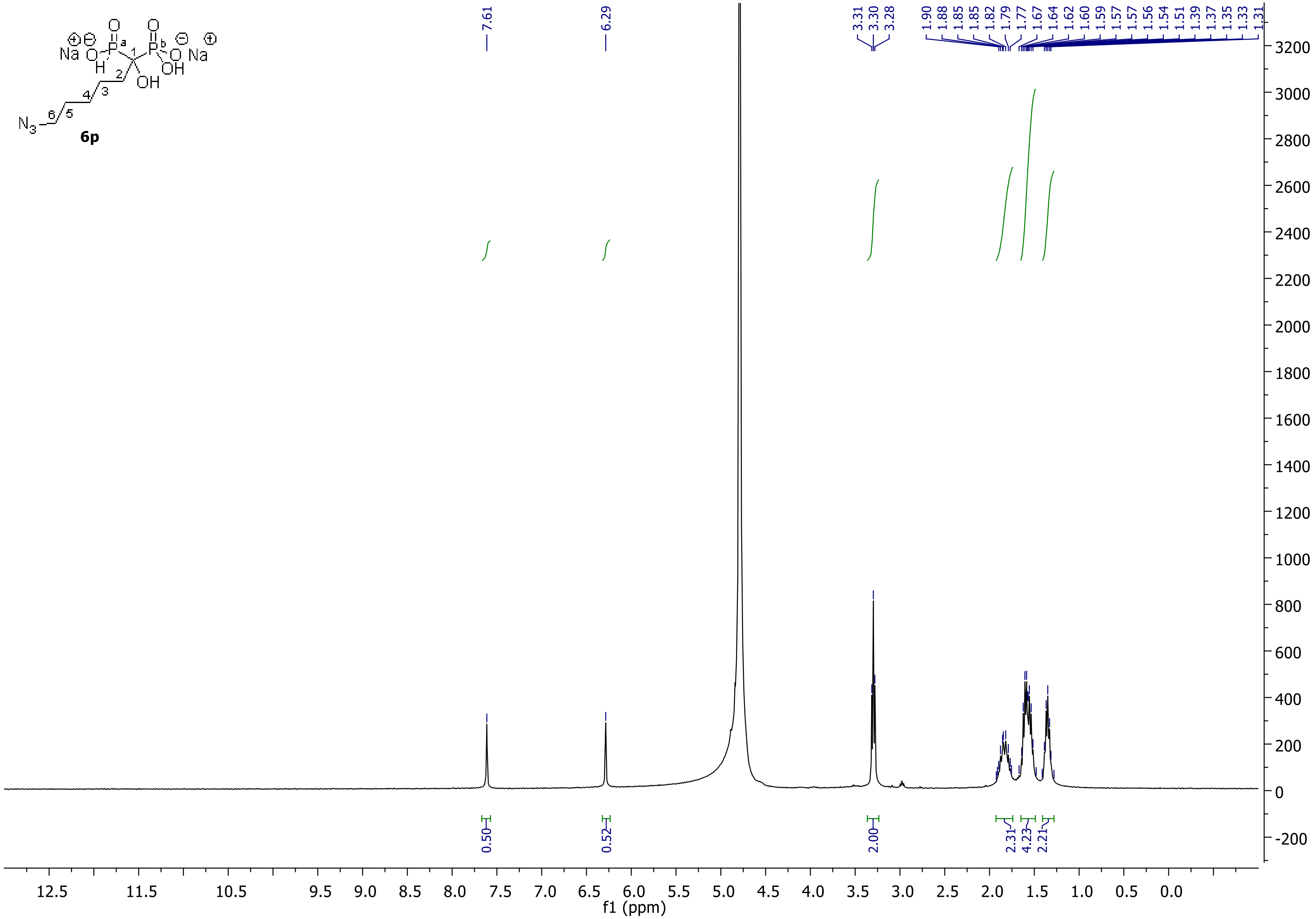

Figure S46: ^{1}H NMR (400 MHz, D_2O) spectrum of 1-hydroxy-1-(5-azidopentyl)methane-1,1-bis(*H*-phosphinylphosphonate) disodium salt 6p

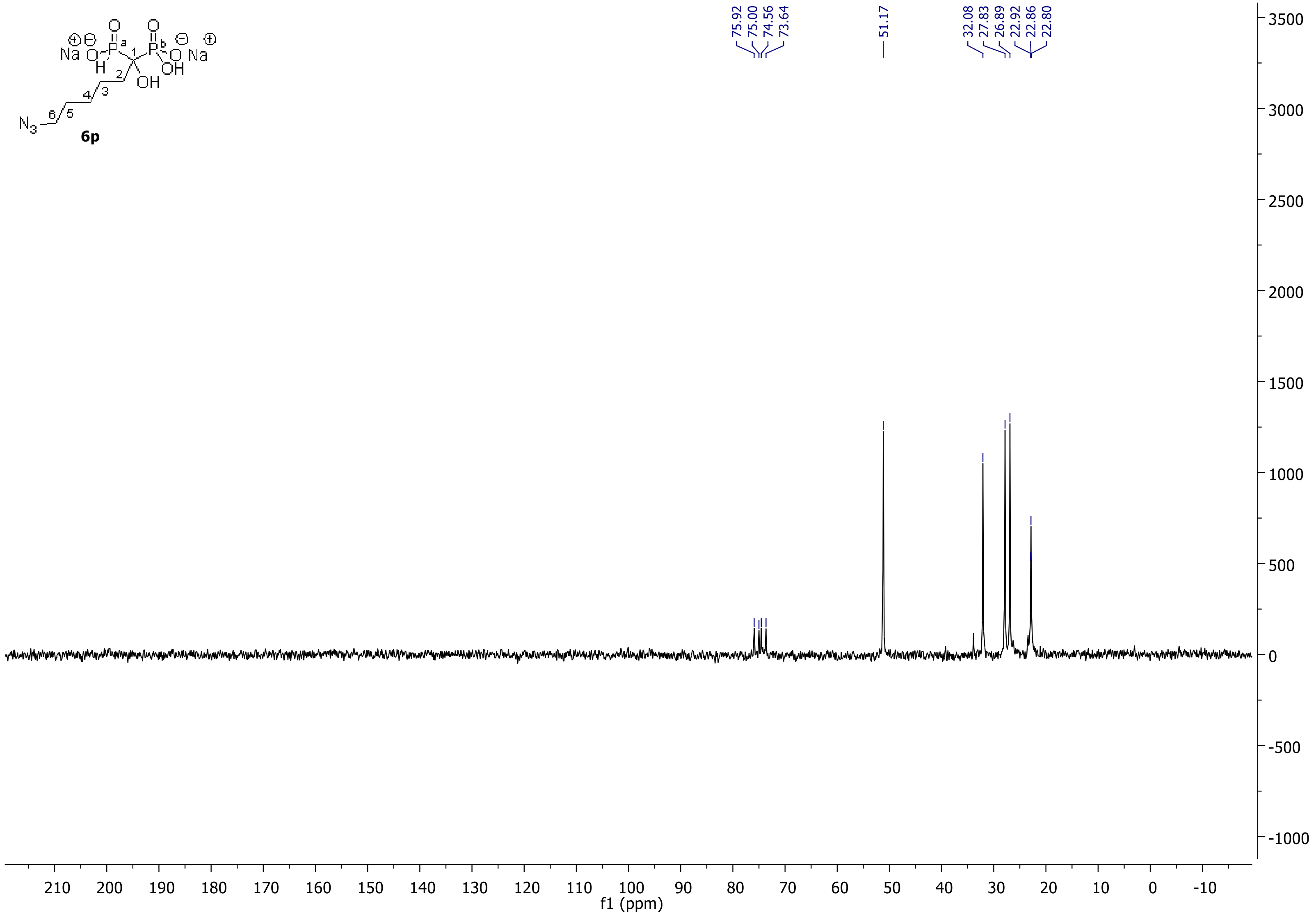

Figure S47 : ^{13}C NMR (101 MHz, D_2O) spectrum of 1-hydroxy-1-(5-azidopentyl)methane-1,1-bis(*H*-phosphinylphosphonate) disodium salt 6p

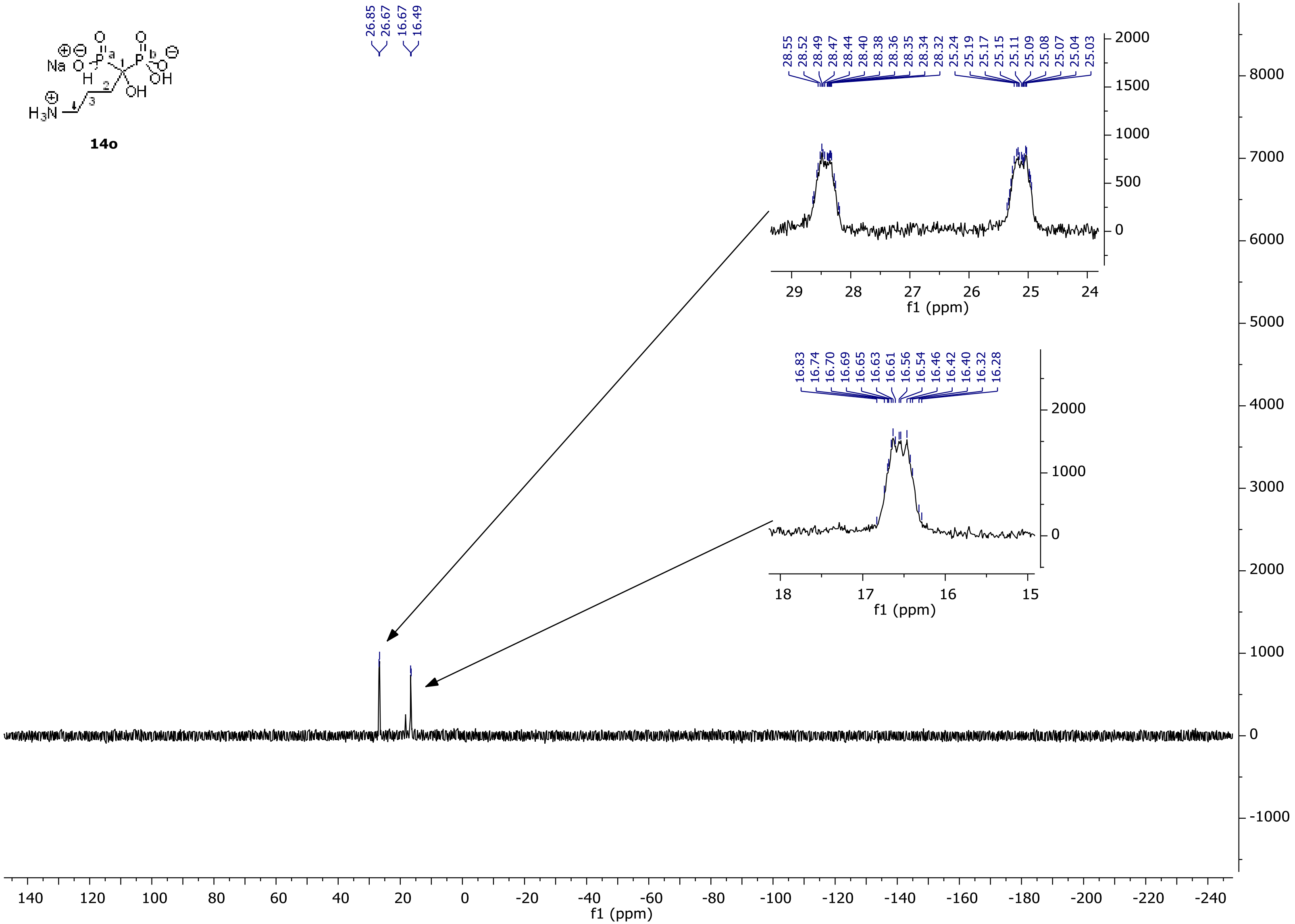

Figure S48: $^{31}P\{^{1}H\}$ and ^{31}P NMR (162 MHz, D_2O) spectra of 1-hydroxy-1-(3-aminopropyl)methane-1,1-bis(*H*-phosphinylphosphonate) disodium salt 14o

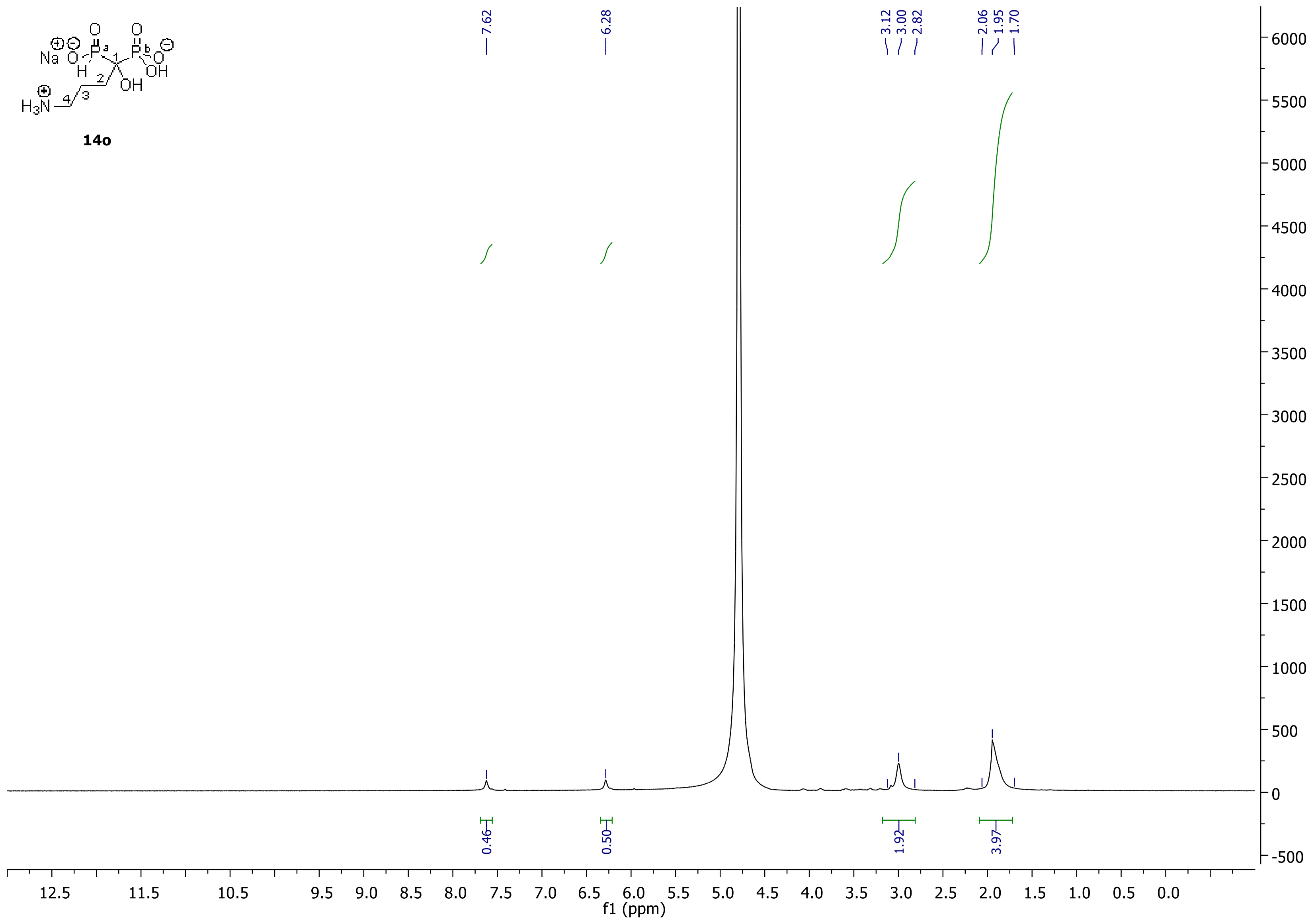

Figure S49: ^{1}H NMR (400 MHz, D_2O) spectrum of 1-hydroxy-1-(3-aminopropyl)methane-1,1-bis(*H*-phosphinylphosphonate) disodium salt 14o

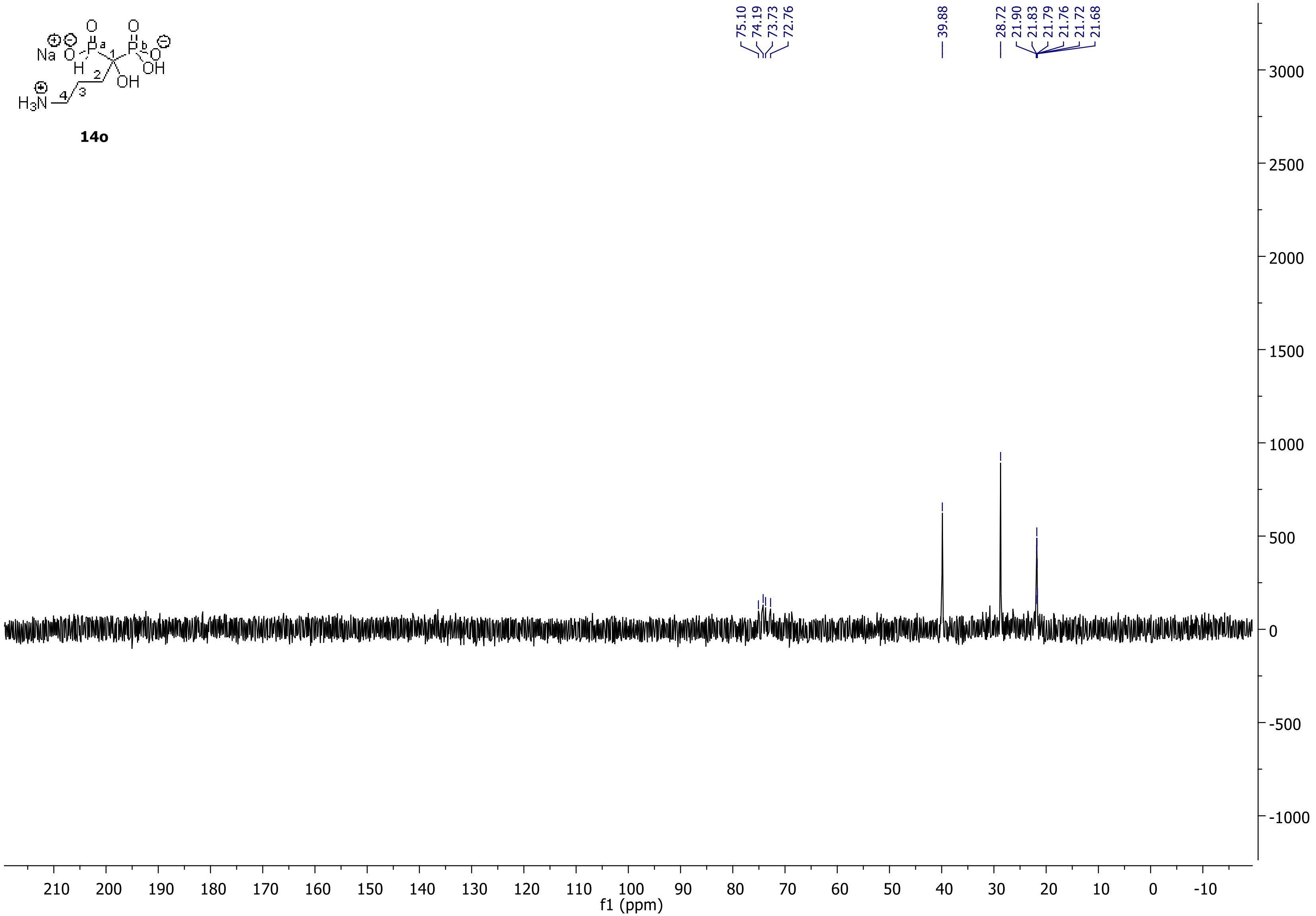

Figure S50: ^{13}C NMR (101 MHz, D_2O) spectrum of 1-hydroxy-1-(3-aminopropyl)methane-1,1-bis(*H*-phosphinylphosphonate) disodium salt 14o

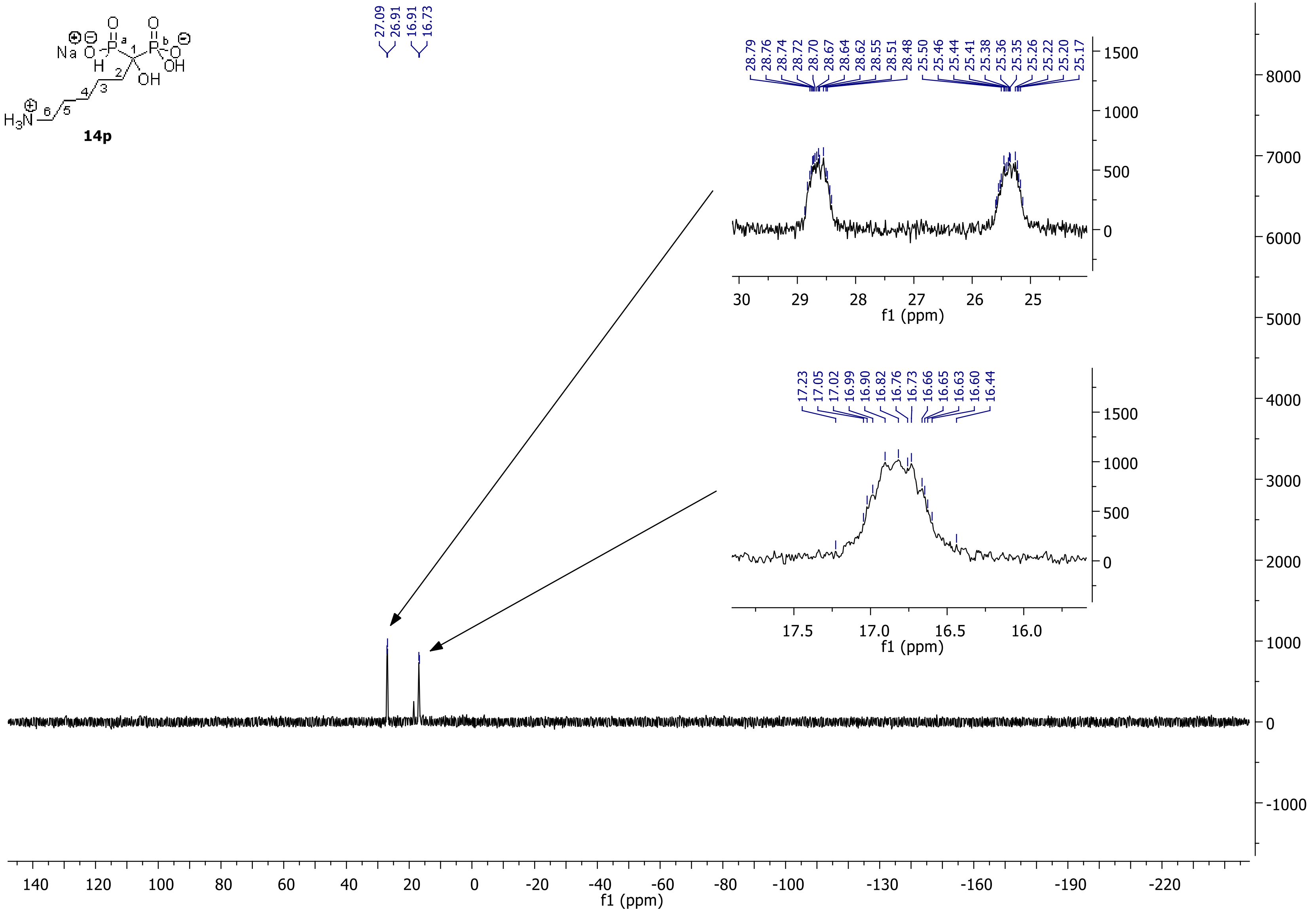

Figure S51: $^{31}P\{^{1}H\}$ and ^{31}P NMR (162 MHz, D_2O) spectra of 1-hydroxy-1-(5-aminopentyl)methane-1,1-bis(*H*-phosphinylphosphonate) disodium salt 14p

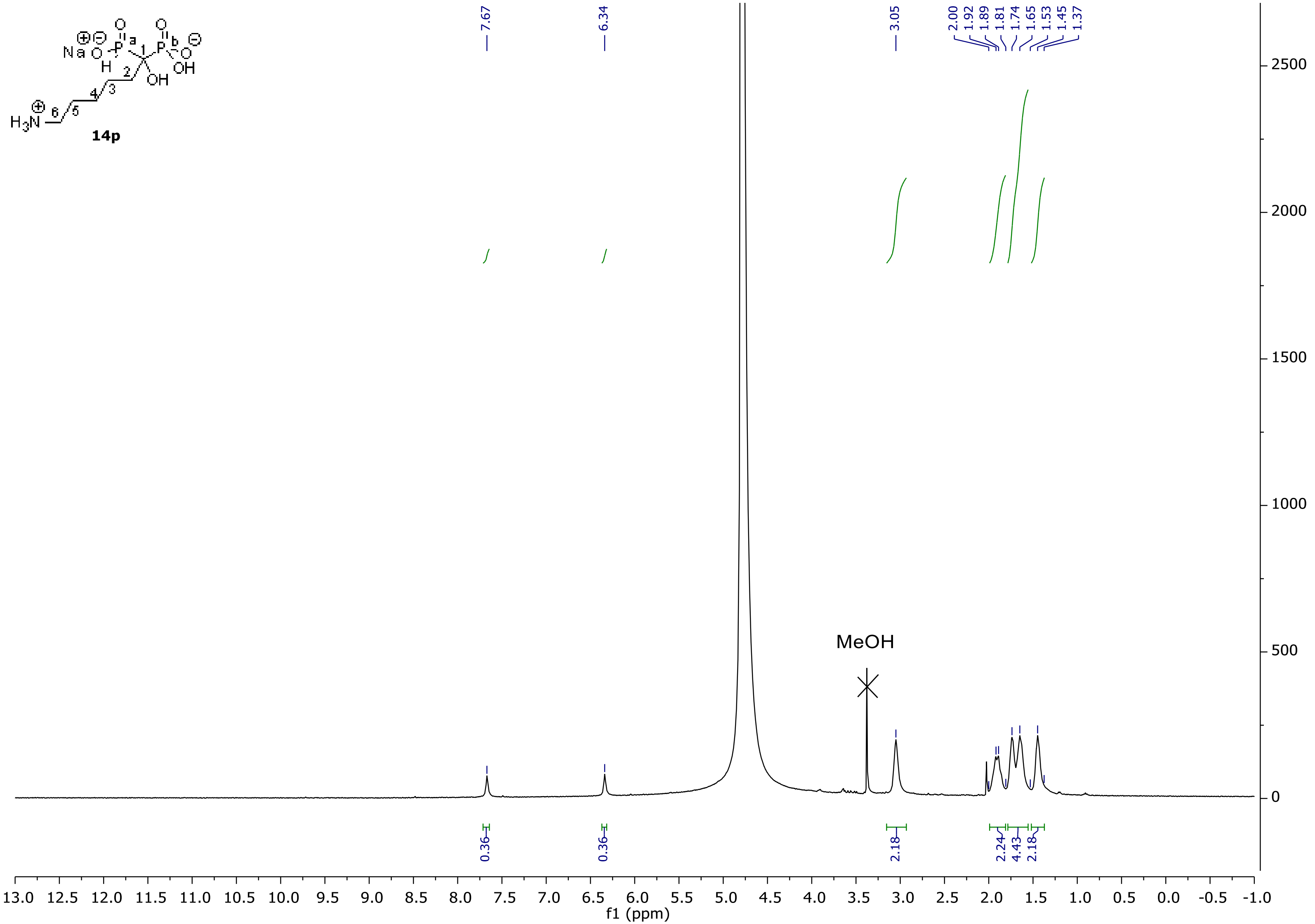

Figure S52: ^{1}H NMR (400 MHz, D_2O) spectrum of 1-hydroxy-1-(5-aminopentyl)methane-1,1-bis(*H*-phosphinylphosphonate) disodium salt 14p

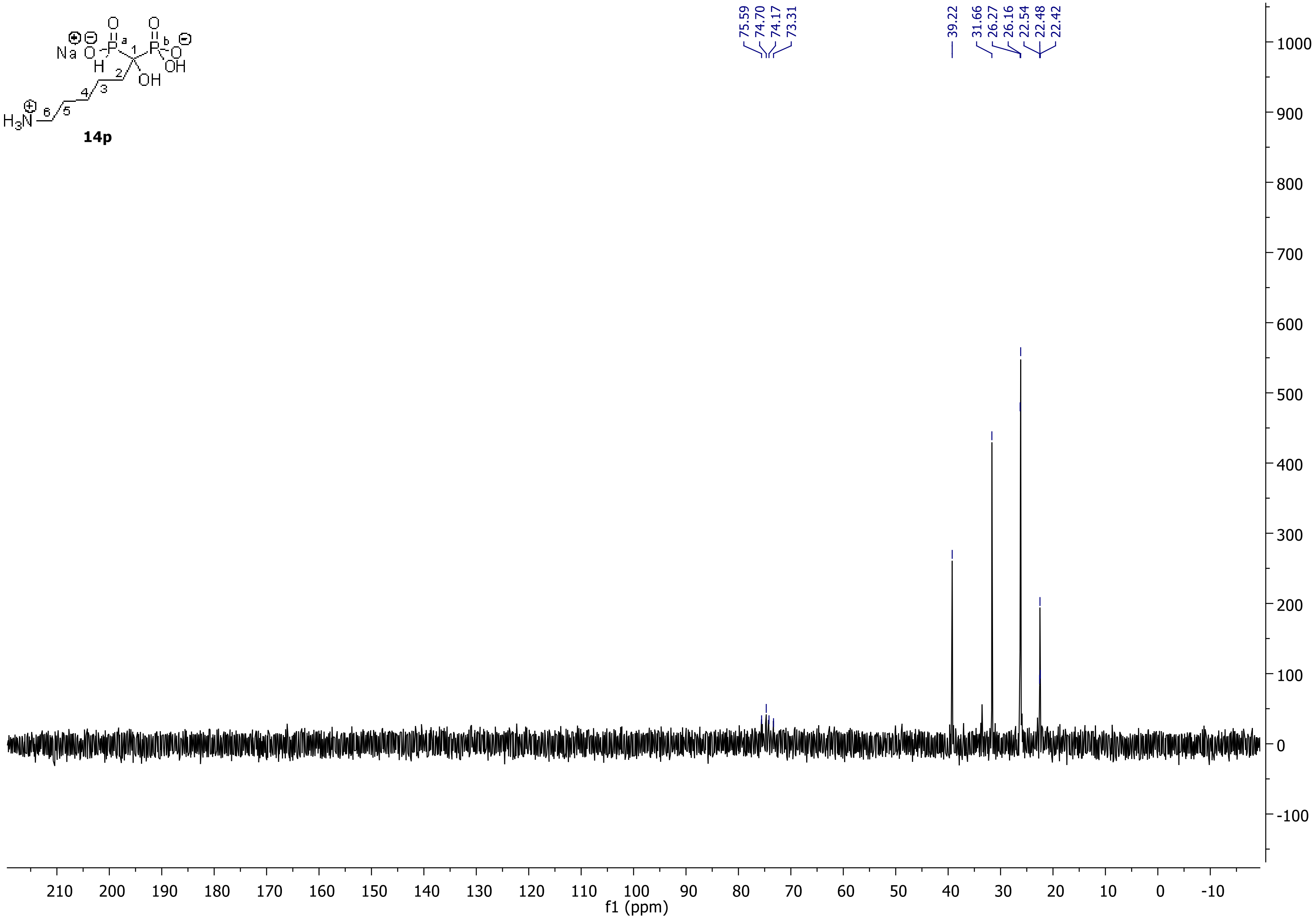

Figure S53: ^{13}C NMR (101 MHz, D_2O) spectrum of 1-hydroxy-1-(5-aminopentyl)methane-1,1-bis(*H*-phosphinylphosphonate) disodium salt 14p
